# Supplementary material for: Ethnomedicinal documentation, phytochemical characterization, and biological evaluation of the traditional medicinal plants from Swat region of Pakistan
Source: PLoS One. 2025 Aug 21;20(8):e0329735. doi: 10.1371/journal.pone.0329735 (PMC12370205; doi:10.1371/journal.pone.0329735)
Supplement: S1 File — Supplementary tables for ethnomedicinal documentation, phytochemical characterization, and antimicrobial, antibiofilm, and cytotoxicity assays. S1 Table. Survey form for the data and sample collection of traditional medicinal plants from Swat, Pakistan. S2 Table. Quantitative analysis of the proportional distribution of each phytochemical constituent across 17 traditional medicinal plants from Swat, Pakistan. S3 Table. Antimicrobial activities (percent inhibition values of two replicate experiments, mean ± SD) of plant extracts against gram-positive bacteria. S4 Table. Antimicrobial activities (percent inhibition values of two replicate experiments, mean ± SD) of plant extracts against gram-negative bacteria. S5 Table. Antimicrobial activities (percent inhibition values of two replicate experiments, mean ± SD) of plant extracts against fungi. S6 Table. Antibiofilm activity (percent growth inhibition of two replicate experiments, mean ± SD) of plant extracts against Staphylococcus aureus (USA 300). S7 Table. Antibiofilm activity (percent growth inhibition of two replicate experiments, mean ± SD) of plant extracts against Candida albicans (SC 5314). S8 Table. Cytotoxicity (cell viability inhibition of two replicate experiments, mean ± SD) of plant extracts against A549 cell lines. S9 Table. Cytotoxicity (cell viability inhibition of two replicate experiments, mean ± SD) of plant extracts against WI-26 VA4 cell lines. Legend for S3–S9 Tables: No. 1–17 = Plant samples; sub-numbers (−1 to −5) = solvents used (hexane, acetone, ethanol, methanol, water); E1 = Experiment 1; E2 = Experiment 2; M ± SD = Mean ± Standard Deviation. (PDF) [file pone.0329735.s001.pdf]

SUPPORTING INFORMATION

S1 Table. Survey form for the data and sample collection of traditional medicinal plants from Swat, Pakistan.

Survey Form: Swat's Traditional Medicinal Plants

1. Voucher ID: ______________________
2. Local name: ______________________
3. Scientific name : ______________________
4. English name:__________________________
5. Plant family: ___________________________
6. Collection site (village): ____________________
7. GPS coordinates: _____________________
8. Plant parts: ______________________
9. Traditional medicinal uses: ___________________________

Date:________________________

S2 Table. Quantitative analysis of the proportional distribution of each phytochemical constituent across 17 traditional medicinal plants from Swat, Pakistan.

Compound	− *(Absence)*			+ *(Low presence)*			++ *(Moderate presence)*			+++ *(High presence)*		
	**Count**	**Percentage**	**Proportion**	**Count**	**Percentage**	**Proportion**	**Count**	**Percentage**	**Proportion**	**Count**	**Percentage**	**Proportion**
Alkaloids	0	0%	0.00	3	17.6%	0.18	10	58.8%	0.59	4	23.5%	0.24
Flavonoids	0	0%	0.00	3	17.6%	0.18	11	64.7%	0.65	3	17.6%	0.18
Phenols	1	5.9%	0.06	6	35.3%	0.35	6	35.3%	0.35	4	23.5%	0.24
Steroids	2	11.8%	0.12	7	41.2%	0.41	6	35.3%	0.35	2	11.8%	0.12
Terpenoids	0	0%	0.00	5	29.4%	0.29	6	35.3%	0.35	6	35.3%	0.35
Coumarins	1	5.9%	0.06	9	52.9%	0.53	6	35.3%	0.35	1	5.9%	0.06
Tannins	0	0%	0.00	3	17.6%	0.18	8	47.1%	0.47	6	35.3%	0.35
Saponins	2	11.8%	0.12	5	29.4%	0.29	4	23.5%	0.24	6	35.3%	0.35
Chalcones	6	35.3%	0.35	5	29.4%	0.29	5	29.4%	0.29	1	5.9%	0.06
Quinones	0	0%	0.00	3	47.1%	0.47	8	35.3%	0.35	6	17.6%	0.18

S3 Table. Antimicrobial activities (percent inhibition values of two replicate experiments, mean ± SD) of plant extracts against gram-positive bacteria.

Herb No.	Plant name	*Staphylococcus aureus*			*Staphylococcus epidermidis*			*Micrococcus luteus*			*Streptococcus faecalis*			*Enterococcus faecalis*		
		**E1**	**E2**	**M±SD**	**E1**	**E2**	**M±SD**	**E1**	**E2**	**M±SD**	**E1**	**E2**	**M±SD**	**E1**	**E2**	**M±SD**
1.1	***Debreagesia salicifolia***	19	31	25±6	38	44	41±3	43	47	45±2	1	1	1±0	48	42	45±3
1.2		44	28	36±8	46	22	34±12	70	82	76±6	7	-1	3±4	39	55	47±8
1.3		36	40	38±2	45	35	40±5	67	59	63±4	40	26	33±7	70	66	68±2
1.4		57	83	70±13	56	42	49±7	81	91	86±5	60	66	63±3	71	59	65±6
1.5		-34	-20	-27±7	-51	-47	-49±2	-26	-30	-28±2	40	56	48±8	1	3	2±1
2.1	***Ajuga bracteosa***	39	33	36±3	2	10	6±4	40	38	39±1	-5	-9	-7±2	-10	-16	-13±3
2.2		27	19	23±4	20	12	16±4	44	48	46±2	-17	7	-5±12	-12	-48	-30±18
2.3		9	11	10±1	5	7	6±1	53	55	54±1	-13	1	-6±7	-62	-66	-64±2
2.4		-23	-17	-20±3	-18	-12	-15±3	-3	-5	-4±1	-13	-25	-19±6	-30	-18	-24±6
2.5		-11	-21	-16±5	27	13	20±7	-51	-79	-65±14	-105	-65	-85±20	-33	-37	-35±2
3.1	***Berberis lyceum***	5	-3	1±4	-13	-17	-15±2	52	58	55±3	-4	-2	-3±1	43	43	43±0
3.2		82	112	97±15	31	41	36±5	91	107	99±8	22	16	19±3	0	14	7±7
3.3		103	89	96±7	39	23	31±8	104	94	99±5	10	6	8±2	-9	-3	-6±3
3.4		87	113	100±13	16	42	29±13	103	95	99±4	-4	10	3±7	-5	-15	-10±5
3.5		6	10	8±2	54	72	63±9	27	41	34±7	-63	-55	-59±4	-10	-8	-9±1
4.1	***Aesculus indica***	53	39	46±7	43	37	40±3	55	65	60±5	22	4	13±9	13	23	18±5
4.2		71	65	68±3	36	36	36±0	87	75	81±6	3	1	2±1	19	43	31±12
4.3		24	12	18±6	13	11	12±1	68	100	84±16	-3	1	-1±2	13	1	7±6
4.4		55	71	63±8	23	15	19±4	88	114	101±13	24	20	22±2	37	41	39±2
4.5		62	56	59±3	80	82	81±1	77	89	83±6	81	73	77±4	5	-35	-15±20
5.1	***Calotropis procera***	-16	-8	-12±4	-4	-2	-3±1	26	32	29±3	-25	-9	-17±8	-17	-25	-21±4
5.2		85	105	95±10	17	11	14±3	33	45	39±6	-4	6	1±5	-56	-124	-90±34
5.3		103	89	96±7	10	10	10±0	50	50	50±0	-12	-4	-8±4	60	74	60±7
5.4		14	18	16±2	5	15	10±5	32	26	29±3	-1	-13	-7±6	34	36	35±1
5.5		-35	-21	-28±7	99	81	90±9	-24	-38	-31±7	-2	-6	-4±2	-52	-68	-60±8

6.1	***Plantago major***	16	8	12±4	9	13	11±2	33	39	36±3	-24	6	-9±15	-50	-56	-53±3
6.2		50	52	51±1	34	24	29±5	68	66	67±1	-7	-19	-13±6	-69	-57	-63±6
6.3		12	22	17±5	4	18	11±7	52	46	49±3	-65	-19	-42±23	-112	-130	-121±9
6.4		-30	-24	-27±3	21	19	20±1	33	49	41±8	-28	-52	-40±12	-96	-146	-121±25
6.5		-30	-18	-24±6	4	-2	1±3	-30	-24	-27±3	9	15	12±3	-72	-44	-58±14
7.1	***Origanum vulgare***	108	92	100±8	23	13	18±5	53	51	52±1	-1	1	0±1	-14	-12	-13±1
7.2		98	108	103±5	37	23	30±7	32	32	32±0	7	13	10±3	36	26	31±5
7.3		52	70	61±9	8	26	17±9	84	118	101±17	3	-5	-1±4	-39	-55	-47±8
7.4		-112	-78	-95±17	-4	-2	-3±1	75	81	78±3	-1	11	5±6	-26	-20	-23±3
7.5		-5	-11	-8±3	-5	-21	-13±8	-32	-48	-40±8	-159	-111	-135±24	29	41	35±6
8.1	***Dysphania ambrosioides***	23	33	28±5	24	16	20±4	51	55	53±2	3	-1	1±2	-7	11	2±9
8.2		93	105	99±6	41	27	34±7	76	90	83±7	-15	-1	-8±7	112	96	104±8
8.3		92	108	100±8	28	32	30±2	78	70	74±4	18	8	13±5	7	13	10±3
8.4		-30	-24	-27±3	9	9	90±0	33	37	35±2	-17	-11	-14±3	-50	-60	-55±5
8.5		-53	-37	-45±8	9	5	7±2	-70	-80	-75±5	-154	-112	-133±21	43	35	39±4
9.1	***Ziziphus oxyphylla***	77	53	65±12	30	32	31±1	59	61	60±1	16	4	10±6	-61	-75	-68±7
9.2		95	109	102±7	50	34	42±8	74	86	80±6	13	9	11±2	74	78	76±2
9.3		86	112	99±13	24	44	34±10	59	59	59±0	25	23	24±1	94	106	100±6
9.4		-27	-19	-23±4	6	8	7±1	31	39	35±4	7	7	7±0	104	86	95±9
9.5		-21	-19	-20±1	-64	-106	-85±21	6	10	8±2	-83	-71	-77±6	48	44	46±2
10.1	***Thymus linearis***	32	22	27±5	8	4	6±2	55	53	54±1	6	10	8±2	-32	-20	-26±6
10.2		80	80	80±0	38	50	44±6	85	101	93±8	6	18	12±6	-75	-81	-78±3
10.3		92	104	98±6	39	43	41±2	89	85	87±2	9	1	5±4	91	59	75±16
10.4		93	101	97±4	38	38	38±0	88	90	89±1	4	18	11±7	73	59	66±7
10.5		47	41	44±3	5	3	4±1	-28	-36	-32±4	82	96	89±7	-3	3	0±3
11.1	***Mentha longifolia***	92	110	101±9	8	16	12±4	52	46	49±3	13	-5	4±9	-13	5	-4±9
11.2		84	110	97±13	28	44	36±8	91	103	97±6	27	27	27±0	93	83	88±5
11.3		77	121	99±22	30	24	27±3	88	82	85±3	15	17	16±1	79	81	80±1
11.4		-43	-45	-44±1	-7	-19	-13±6	45	43	44±1	-3	-3	-3±0	-73	-107	-90±17
11.5		19	25	22±3	25	11	18±7	-38	-48	-43±5	-5	-3	-4±1	-66	-74	-70±4
12.1	***Punica granatum***	-4	6	1±5	33	37	35±2	51	57	54±3	15	7	11±4	-29	-41	-35±6

12.2		57	85	71±14	56	32	44±12	72	106	89±17	37	43	40±3	88	104	96±8
12.3		75	65	70±5	32	46	39±7	82	94	88±6	56	60	58±2	29	33	31±2
12.4		97	79	88±9	67	55	61±6	90	86	88±2	62	58	60±2	81	89	85±4
12.5		65	77	71±6	88	96	92±4	60	60	60±0	27	35	31±4	26	22	24±2
13.1	***Juglans regia***	85	117	101±16	19	15	17±2	29	47	38±9	19	29	24±5	-66	-82	-74±8
13.2		96	86	91±5	97	97	97±0	132	108	120±12	107	95	101±6	120	172	146±26
13.3		95	89	92±3	104	148	126±22	88	90	89±1	79	93	86±7	87	97	92±5
13.4		95	111	103±8	92	100	96±4	95	103	99±4	99	105	102±3	97	101	99±2
13.5		83	107	95±12	99	93	96±3	54	60	57±3	-7	-3	-5±2	10	10	10±0
14.1	***Salvia moorcroftiana***	92	96	94±2	-10	-8	-9±1	38	46	42±4	13	5	9±4	-141	-205	-173±32
14.2		93	107	100±7	41	31	36±5	51	67	59±8	11	9	10±1	-37	-47	-42±5
14.3		-5	-9	-7±2	21	21	21±0	69	61	65±4	5	5	5±0	-41	-37	-39±2
14.4		-92	-30	-61±31	17	5	11±6	49	43	46±3	-9	1	-4±5	-153	-171	-162±9
14.5		-11	-15	-13±2	94	78	86±8	-83	-47	-65±18	81	75	78±3	28	32	30±2
15.1	***Artemesia maritima***	91	105	98±7	21	13	17±4	57	63	60±3	8	18	13±5	-66	-84	-75±9
15.2		106	94	100±6	46	42	44±2	84	68	76±8	13	27	20±7	98	78	88±10
15.3		89	105	97±8	79	87	83±4	65	69	67±2	22	18	20±2	44	56	50±6
15.4		84	110	97±13	29	41	35±6	66	72	69±3	42	42	42±0	78	74	76±2
15.5		-27	-19	-23±4	-5	-7	-6±1	-22	-36	-29±7	-17	-11	-14±3	-62	-74	-68±6
16.1	***Mentha spicata***	85	115	100±15	39	25	32±7	47	35	41±6	29	15	22±7	-152	-172	-162±10
16.2		81	121	101±20	28	26	27±1	78	80	79±1	6	22	14±8	-2	4	1±3
16.3		104	86	95±9	37	27	32±5	58	68	63±5	13	19	16±3	-59	-69	-64±5
16.4		86	112	99±13	32	26	29±3	56	40	48±8	24	12	18±6	-44	-42	-43±1
16.5		-22	-8	-15±7	1	3	2±1	-34	-42	-38±4	81	69	75±6	-98	-82	-90±8
17.1	***Nasturtium officinale***	96	104	100±4	24	40	32±8	31	35	33±2	22	22	22±0	-128	-172	-150±22
17.2		96	116	106±10	83	81	82±1	62	92	77±15	21	25	23±2	78	76	77±1
17.3		93	93	93±0	27	29	28±1	42	56	49±7	24	16	20±4	61	73	67±6
17.4		73	59	66±7	78	68	73±5	43	39	41±2	16	2	9±7	62	50	56±6
17.5		-25	-35	-30±5	-32	-34	-33±1	-14	-24	-19±5	6	4	5±1	34	34	34±0

Legend. No. 1-17: Plants mentioned: sub-number (-1 to -5): hexane, acetone, ethanol, methanol, water; E1: Experiment 1; E2: Experiment 2; M±SD: Mean ± Standard Deviation.

S4 Table. Antimicrobial activities (percent inhibition values of two replicate experiments, mean ± SD) of plant extracts against gram-negative bacteria.

Herb No.	Plant name	*Escherichia coli*			*Brevundimonas diminuta*			*Salmonella enteritidis*			*Enterobacter aerogenes*			*Acinetobacter baumannii*			*Shigella flexneri*			*Shigella sonnei*			*Aeromonas hydrophila*			*Pseudomonas aeruginosa*		
		E1	E2	M±SD	E1	E2	M±SD	E1	E2	M±SD	E1	E2	M±SD	E1	E2	M±SD	E1	E2	M±SD	E1	E2	M±SD	E1	E2	M±SD	E1	E2	M±SD
1.1	*Debreagesia salicifolia*	33	13	23±10	105	89	97±8	46	38	42±4	21	11	16±5	7	11	9±2	43	41	42±1	38	32	35±3	99	95	97±2	7	5	6±1
1.2		13	17	15±2	111	105	108±3	26	30	28±2	23	19	21±2	-8	-18	-13±5	27	19	23±4	-2	0	-1±1	94	104	99±5	-56	-32	-44±12
1.3		33	19	26±7	90	92	91±1	14	20	17±3	5	3	4±1	-13	-15	-14±1	4	-2	1±3	60	68	64±4	96	102	99±3	-49	-41	-45±4
1.4		35	51	43±8	94	106	100±6	38	36	37±1	71	77	74±3	29	41	35±6	11	13	12±1	-4	8	2±6	91	105	98±7	4	8	6±2
1.5		-44	-50	-47±3	-134	-180	-157±23	-35	-25	-30±5	-65	-83	-74±9	-13	-3	-8±5	57	69	63±6	-125	-193	-159±34	-81	-109	-95±14	-127	-109	-118±9
2.1	*Ajuga bracteosa*	5	-3	1±4	94	102	98±4	25	21	23±2	26	26	26±0	-17	-23	-20±3	-7	-1	-4±3	-43	-57	-50±7	76	60	68±8	32	28	30±2
2.2		30	16	23±7	105	95	100±5	28	22	25±3	17	21	19±2	3	7	5±2	-12	-2	-7±5	22	14	18±4	92	88	90±2	-2	4	1±3
2.3		16	26	21±5	93	85	89±4	22	10	16±6	21	27	24±3	-10	-2	-6±4	-26	-8	-17±9	19	23	21±2	100	94	97±3	-8	-6	-7±1
2.4		9	3	6±3	63	57	60±3	2	16	9±7	14	4	9±5	-43	-55	-49±6	15	9	12±3	-6	-4	-5±1	8	0	4±4	-9	-21	-15±6
2.5		-73	-87	-80±7	-107	-137	-122±15	13	15	14±1	-42	-56	-49±7	-40	-42	-41±1	21	15	18±3	-38	-52	-45±7	-63	-75	-69±6	-145	-109	-127±18
3.1	*Berberis lyceum*	-10	14	2±12	19	25	22±3	7	11	9±2	22	28	25±3	5	17	11±6	19	9	14±5	14	8	11±3	1	3	2±1	10	16	13±3
3.2		45	39	42±3	93	107	100±7	30	16	23±7	25	27	26±1	16	20	18±2	28	26	27±1	41	39	40±1	9	5	7±2	69	59	64±5
3.3		45	33	39±6	16	26	21±5	15	23	19±4	29	25	27±2	45	39	42±3	28	28	28±0	88	94	91±3	19	7	13±6	44	42	43±1
3.4		33	35	34±1	103	85	94±9	18	20	19±1	18	12	15±3	6	20	13±7	29	31	30±1	52	42	47±5	42	38	40±2	36	24	30±6
3.5		-39	-67	-53±14	-21	-27	-24±3	71	71	71±0	54	52	53±1	41	45	43±2	22	30	26±4	17	15	16±1	-78	-88	-83±5	-78	-60	-69±9
4.1	*Aesculus indica*	43	29	36±7	107	125	116±9	32	28	30±2	3	11	7±4	21	21	21±0	42	24	33±9	34	44	39±5	94	106	100±6	19	33	26±7
4.2		36	30	33±3	75	65	70±5	33	21	27±6	18	8	13±5	-12	-20	-16±4	-3	1	-1±2	59	63	61±2	104	96	100±4	-20	-16	-18±2
4.3		23	35	29±6	78	76	77±1	14	12	13±1	24	18	21±3	-31	-27	-29±2	2	4	3±1	53	55	54±1	103	97	100±3	-47	-23	-35±12
4.4		11	15	13±2	104	98	101±3	14	18	16±2	14	18	16±2	-9	-3	-6±3	19	13	16±3	48	36	42±6	105	93	99±6	-32	-46	-39±7
4.5		-30	-76	-53±23	22	36	29±7	23	35	29±6	-23	-31	-27±4	-20	-28	-24±4	-9	1	-4±5	-31	-45	-38±7	-17	-7	-12±5	-50	-54	-52±2
5.1	*Calotropis procera*	1	-17	-8±9	86	82	84±2	-21	-3	-21±9	10	8	9±1	-9	-15	-12±3	1	3	2±1	27	21	24±3	109	95	102±7	-6	-12	-9±3
5.2		30	26	28±2	94	92	93±1	2	0	1±1	10	10	10±0	2	4	3±1	16	8	12±4	-29	-31	-30±1	106	98	102±4	2	6	4±2
5.3		3	5	4±1	77	77	77±0	5	3	4±1	0	-8	-4±4	-21	-31	-26±5	8	20	14±6	-49	-39	-44±5	106	94	100±6	-11	-7	-9±2
5.4		13	7	10±3	105	113	109±4	56	64	60±4	12	8	10±2	-33	-29	-31±2	20	24	22±2	35	19	27±8	95	101	98±3	-70	-58	-64±6
5.5		-61	-101	-80±20	-163	-195	-179±16	66	72	69±3	-78	-94	-86±8	72	90	81±9	82	72	77±5	-65	-103	-84±19	-23	-33	-28±5	-87	-77	-82±5
6.1		7	7	7±0	92	78	85±7	31	21	26±5	23	29	26±3	2	6	4±2	12	26	19±7	27	19	23±4	108	92	100±8	35	31	33±2

6.2	***Plantago major***	17	19	18±1	85	91	88±3	19	15	17±2	9	3	6±3	3	-3	0±3	12	16	14±2	-38	-56	-47±9	77	83	80±3	-32	-30	-31±1
6.3		14	0	7±7	89	99	94±5	2	6	4±2	5	-3	1±4	-23	-31	-27±4	8	6	7±1	-50	-44	-47±3	90	102	96±6	-34	-30	-32±2
6.4		7	11	9±2	76	82	79±3	1	-3	-1±2	2	-8	-3±5	-23	-25	-24±1	13	5	9±4	-59	-47	-53±6	97	113	105±8	-60	-44	-52±8
6.5		-74	-92	-83±9	-113	-153	-133±20	-33	-23	-28±5	57	71	64±7	80	80	80±0	-40	-34	-37±3	-99	-125	-112±13	-95	-119	-107±12	-115	-135	-125±10
7.1	***Origanum vulgare***	33	23	28±5	50	38	44±6	82	96	89±7	4	8	6±2	-2	-4	-3±1	-2	12	5±7	57	45	51±6	103	89	96±7	8	10	9±1
7.2		-4	-8	-6±2	41	45	43±2	17	29	23±6	-7	-9	-8±1	22	32	27±5	7	11	9±2	6	2	4±2	97	93	95±2	-33	-27	-30±3
7.3		-4	-2	-3±1	39	25	32±7	16	16	16±0	14	14	14±0	-12	-8	-10±2	21	9	15±6	-4	2	-1±3	107	119	113±6	-35	-43	-39±4
7.4		9	21	15±6	84	66	75±9	10	20	15±5	6	10	8±2	-8	-14	-11±3	14	10	12±2	-2	-10	-6±4	108	94	101±7	-59	-43	-51±8
7.5		-76	-98	-87±11	-100	-136	-118±18	63	71	67±4	-73	-101	-87±14	-42	-46	-44±2	-73	-49	-61±12	-86	-116	-101±15	-81	-101	-91±10	-142	-114	-128±14
8.1	***Dysphania ambrosioides***	2	2	2±0	77	89	83±6	23	15	19±4	12	6	9±3	-20	-18	-19±1	7	9	8±1	25	21	23±2	59	53	56±3	5	7	6±1
8.2		15	21	18±3	97	89	93±4	29	31	30±1	-8	-18	-13±5	15	23	19±4	9	15	12±3	56	52	54±2	92	102	97±5	-20	-24	-22±2
8.3		1	11	6±5	96	90	93±3	9	15	12±3	9	13	11±2	-6	4	-1±5	8	10	9±1	45	47	46±1	94	104	99±5	-34	-32	-33±1
8.4		15	21	18±3	88	78	83±5	24	10	17±7	10	2	6±4	-5	-19	-12±7	5	-7	-1±6	14	18	16±2	38	34	36±2	-31	-19	-25±6
8.5		-52	-82	-67±15	-139	-157	-148±9	84	96	90±6	-82	-98	-90±8	93	105	99±6	77	85	81±4	-81	-97	-89±8	-96	-108	-102±6	-131	-107	-119±12
9.1	***Ziziphus oxyphylla***	36	18	27±9	30	24	27±3	28	32	30±2	6	10	8±2	2	-4	-1±3	16	20	18±2	20	14	17±3	120	98	109±11	24	20	22±2
9.2		20	8	14±6	112	96	104±8	23	25	24±1	30	24	27±3	23	23	23±0	-6	2	-2±4	8	6	7±1	105	95	100±5	22	14	18±4
9.3		17	21	19±2	87	91	89±2	24	24	24±0	23	19	21±2	20	24	22±2	-5	-11	-8±3	6	2	4±2	99	97	98±1	10	8	9±1
9.4		0	-20	-10±10	82	82	82±0	15	9	12±3	2	4	3±1	-4	-10	-7±3	11	11	11±0	-2	-4	-3±1	94	100	97±3	-16	-8	-12±4
9.5		-48	-56	-52±4	-112	-138	-125±13	-23	-11	-17±6	-41	-73	-57±16	-43	-51	-47±4	-52	-36	-44±8	4	8	6±2	-68	-86	-77±9	-147	-127	-137±10
10.1	***Thymus linearis***	-12	-22	-17±5	85	73	79±6	-2	-6	-4±2	1	11	6±5	16	32	24±8	18	22	20±2	29	25	27±2	61	51	56±5	50	46	48±2
10.2		23	9	16±7	99	109	104±5	41	35	38±3	7	11	9±2	4	6	5±1	10	8	9±1	39	33	36±3	99	93	96±3	-64	-38	-51±13
10.3		1	13	12±1	118	138	128±10	21	29	25±4	11	9	10±1	12	4	8±4	16	16	16±0	51	51	51±0	97	105	101±4	-17	-3	-10±7
10.4		21	13	17±4	98	104	101±3	12	22	17±5	4	4	4±0	-14	-28	-21±7	7	13	10±3	25	21	23±2	99	111	105±6	-40	-36	-38±2
10.5		-76	-120	-98±22	-58	-80	-69±11	53	55	54±1	-62	-86	-74±12	76	86	81±5	107	147	127±20	-47	-63	-55±8	-99	-115	-107±8	-149	-127	-138±11
11.1	***Mentha longifolia***	-2	10	4±6	86	76	81±5	7	-3	2±5	2	6	4±2	3	-5	-1±4	16	12	14±2	32	34	33±1	98	92	95±3	23	35	29±6
11.2		33	19	26±7	99	117	108±9	23	29	26±3	-12	-18	-15±3	32	42	37±5	36	22	29±7	20	10	15±5	104	102	103±1	-45	-51	-48±3
11.3		11	3	7±4	63	67	65±2	17	5	11±6	-14	-28	-21±7	59	55	57±2	30	24	27±3	-8	-14	-11±3	96	104	100±4	-44	-54	-49±5
11.4		-11	1	-5±6	90	82	86±4	23	15	19±4	17	21	19±2	-36	-38	-37±1	21	23	22±1	4	2	3±1	89	85	87±2	-33	-15	-24±9
11.5		-45	-115	-80±35	-106	-110	-108±2	-29	-25	-27±2	-64	-90	-77±13	-43	-37	-40±3	-19	-7	-13±6	-78	-92	-85±7	-66	-80	-73±7	-130	-112	-121±9
12.1	***Punica granatum***	10	6	8±2	49	53	51±2	10	12	11±1	23	7	15±8	47	57	52±5	10	12	11±1	-3	7	2±5	33	39	36±3	25	33	29±4
12.2		22	40	31±9	103	109	106±3	24	28	26±2	14	12	13±1	70	62	66±4	44	54	49±5	40	38	39±1	104	116	110±6	92	82	87±5

12.3		49	39	44±5	100	90	95±5	14	8	11±3	21	9	15±6	74	72	73±1	41	37	39±2	30	26	28±2	114	98	106±8	59	47	53±6
12.4		28	34	31±3	70	82	76±6	30	32	31±1	32	36	34±2	56	56	56±0	48	40	44±4	28	22	25±3	98	106	102±4	53	59	56±3
12.5		-22	-34	-28±6	6	-4	1±5	3	7	5±2	-3	1	-1±2	9	13	11±2	73	83	78±5	18	10	14±4	96	102	99±3	-4	-2	-3±1
13.1	***Juglans regia***	20	14	17±3	97	81	89±8	18	20	19±1	6	8	7±1	2	4	3±1	15	19	17±2	46	52	49±3	104	94	99±5	26	30	28±2
13.2		93	89	91±2	92	88	90±2	44	40	42±2	28	22	25±3	137	109	123±14	92	108	100±8	108	92	100±8	93	89	91±2	74	68	71±3
13.3		83	73	78±5	87	97	92±5	45	55	50±5	31	27	29±2	94	88	91±3	98	108	103±5	103	95	99±4	91	91	91±0	93	101	97±4
13.4		78	84	81±3	87	105	96±9	50	56	53±3	35	25	30±5	82	98	90±8	111	93	102±9	93	107	100±7	105	87	96±9	88	92	90±2
13.5		-15	-45	-30±15	23	35	29±6	1	3	2±1	8	-4	2±6	10	14	12±2	27	23	25±2	-2	-8	-5±3	100	94	97±3	-29	-21	-25±4
14.1	***Salvia moorcroftiana***	-23	-29	-26±3	65	67	66±1	6	4	5±1	5	7	6±1	-8	-22	-15±7	-4	2	-1±3	44	20	32±12	44	48	46±2	10	8	9±1
14.2		-13	-1	-7±6	89	89	89±0	26	26	26±0	1	1	1±0	4	8	6±2	11	13	12±1	24	18	21±3	96	96	96±0	-1	-9	-5±4
14.3		25	19	22±3	79	61	70±9	18	24	21±3	4	0	2±2	0	-2	-1±1	-16	-10	-13±3	42	32	37±5	71	67	69±2	-5	-7	-6±1
14.4		-5	-13	-9±4	106	86	96±10	12	26	19±7	-5	-11	-8±3	5	3	4±1	6	6	6±0	26	30	28±2	94	82	88±6	-44	-28	-36±8
14.5		-58	-90	-74±16	6	10	8±2	65	69	67±2	8	16	12±4	35	45	40±5	80	72	76±4	96	96	96±0	-22	-16	-19±3	17	21	19±2
15.1	***Artemesia maritima***	26	32	29±3	95	85	90±5	22	12	17±5	6	8	7±1	3	9	6±3	23	17	20±3	6	4	5±1	104	96	100±4	21	15	18±3
15.2		4	4	4±0	127	105	116±11	41	29	35±6	-6	-10	-8±2	19	17	18±1	15	29	22±7	83	77	80±3	113	97	105±8	-13	-3	-8±5
15.3		3	5	4±1	101	113	107±6	25	27	26±1	-14	-24	-19±5	20	16	18±2	22	26	24±2	51	51	51±0	95	93	94±1	-3	1	-1±2
15.4		4	-4	0±4	115	119	117±2	15	27	21±6	-25	-27	-26±1	15	11	13±2	27	17	22±5	84	88	86±2	83	91	87±4	-8	-12	-10±2
15.5		-83	-87	-85±2	-101	-127	-114±13	-36	-14	-25±11	84	84	84±0	73	81	77±4	-35	-31	-33±2	-75	-107	-91±16	-82	-110	-96±14	-142	-122	-132±10
16.1	***Mentha spicata***	19	7	13±6	-3	-11	-7±4	34	28	31±3	9	15	12±3	1	3	2±1	17	19	18±1	6	2	4±2	28	20	24±4	23	33	28±5
16.2		-2	2	0±2	81	65	73±8	33	25	29±4	-4	-6	-5±1	-12	-26	-19±7	10	6	8±2	24	30	27±3	58	54	56±2	-4	-2	-3±1
16.3		6	6	6±0	86	78	82±4	27	31	29±2	11	17	14±3	-40	-42	-41±1	15	15	15±0	-18	-22	-20±2	80	78	79±1	-70	-46	-58±12
16.4		3	11	7±4	81	93	87±6	22	34	28±6	16	20	18±2	-19	-13	-16±3	21	27	24±3	-16	-24	-20±4	88	94	91±3	-15	-21	-18±3
16.5		-66	-118	-92±26	-78	-122	-100±22	-27	-19	-23±4	-71	-93	-82±11	-35	-19	-27±8	-36	-26	-31±5	-75	-105	-90±15	-78	-98	-88±10	18	16	17±1
17.1	***Nasturtium officinale***	33	25	29±4	45	37	41±4	11	9	10±1	21	23	22±1	16	18	17±1	-9	-5	-7±2	34	48	41±7	59	55	57±2	41	47	44±3
17.2		8	-2	3±5	107	93	100±7	35	21	28±7	38	34	36±2	23	13	18±5	28	26	27±1	98	104	101±3	96	92	94±2	-14	-6	-10±4
17.3		60	68	64±4	88	94	91±3	30	36	33±3	7	15	11±4	43	49	46±3	21	15	18±3	18	16	17±1	92	102	97±5	5	3	4±1
17.4		31	17	24±7	82	86	84±2	21	13	17±4	29	23	26±3	4	4	4±0	9	3	6±3	76	64	70±6	34	32	33±1	18	24	21±3
17.5		-84	-108	-96±12	-95	-161	-128±33	-29	-27	-28±1	-85	-113	-99±14	-44	-64	-54±10	-21	-19	-20±1	-36	-40	-38±2	-56	-64	-60±4	-109	-93	-101±8

Legend. No. 1-17: Plants mentioned: sub-number (-1 to -5): hexane, acetone, ethanol, methanol, water; E1: Experiment 1; E2: Experiment 2; M±SD: Mean ± Standard Deviation.

S5 Table. Antimicrobial activities (percent inhibition values of two replicate experiments, mean ± SD) of plant extracts against fungi.

Herb No.	*Plant name*	*Candida albicans*			*Candida auris*			*Candida glabrata*			*Candida utilis*			*Candida parapsilosis*			*Saccharomyces cerevisiae*		
		**E1**	**E2**	**M±SD**	**E1**	**E2**	**M±SD**	**E1**	**E2**	**M±SD**	**E1**	**E2**	**M±SD**	**E1**	**E2**	**M±SD**	**E1**	**E2**	**M±SD**
1.1	***Debreagesia salicifolia***	17	11	14±3	35	25	30±5	11	13	12±1	97	99	98±1	6	14	10±4	-18	-26	-22±4
1.2		15	23	19±4	36	44	40±4	71	71	71±0	-55	-27	-41±14	85	81	83±2	-24	-6	-15±9
1.3		23	19	21±2	29	31	30±1	12	18	15±3	1	5	3±2	45	31	38±7	-2	4	1±3
1.4		30	28	29±1	36	42	39±3	15	5	10±5	-41	-53	-47±6	47	47	47±0	23	25	24±1
1.5		-34	-24	-29±5	-11	1	-5±6	-5	-7	-6±1	-4	-12	-8±4	14	20	17±3	1	-13	-6±7
2.1	***Ajuga bracteosa***	8	16	12±4	31	25	28±3	7	15	11±4	-34	-48	-41±7	9	7	8±1	-32	-34	-33±1
2.2		10	12	11±1	43	31	37±6	19	7	13±6	-69	-51	-60±9	41	55	48±7	-17	-25	-21±4
2.3		9	5	7±2	34	36	35±1	11	7	9±2	-62	-28	-45±17	60	72	66±6	-49	-27	-38±11
2.4		12	10	11±1	0	4	2±2	-2	-4	-3±1	-47	-65	-56±9	39	33	36±3	-22	-8	-15±7
2.5		-23	-29	-26±3	12	10	11±1	-2	2	0±2	16	20	18±2	29	25	27±2	-13	-7	-10±3
3.1	***Berberis lyceum***	3	5	4±1	7	-1	3±4	2	4	3±1	-10	-20	-15±5	9	-1	4±5	-13	-25	-19±6
3.2		13	17	15±2	60	76	68±8	89	97	93±4	2	-6	-2±4	95	87	91±4	97	89	93±4
3.3		33	39	36±3	61	65	63±2	98	98	98±0	3	5	4±1	99	95	97±2	94	100	97±3
3.4		53	55	54±1	85	81	83±2	100	96	98±2	25	23	24±1	103	95	99±4	91	101	96±5
3.5		-25	-29	-27±2	34	24	29±5	27	29	28±1	40	50	45±5	20	22	21±1	-5	-11	-8±3
4.1	***Aesculus indica***	22	12	17±5	39	33	36±3	17	11	14±3	9	3	6±3	20	32	26±6	-17	-9	-13±4
4.2		40	28	34±6	16	20	18±2	7	17	12±5	-112	-88	-100±12	43	29	36±7	9	7	8±1
4.3		25	27	26±1	53	55	54±1	10	6	8±2	-26	-42	-34±8	39	43	41±2	82	72	77±5
4.4		45	49	47±2	57	53	55±2	92	100	96±4	8	4	6±2	35	37	36±1	86	102	94±8
4.5		-9	-17	-13±4	65	65	65±0	6	8	7±1	-21	-7	-14±7	24	28	26±2	-1	3	1±2
5.1	***Calotropis procera***	10	14	12±2	43	41	42±1	19	19	19±0	-54	-102	-78±24	68	52	60±8	-42	-30	-36±6
5.2		11	17	14±3	40	46	43±3	13	7	10±3	-117	-53	-85±32	87	79	83±4	-18	-32	-25±7
5.3		8	10	9±1	22	38	30±8	0	8	4±4	-50	-82	-66±16	67	65	66±1	-50	-40	-45±5
5.4		0	13	11±2	32	26	29±3	7	3	5±2	-13	-29	-21±8	73	93	83±10	-5	-9	-7±2
5.5		-17	-31	-24±7	-12	-22	-17±5	7	-3	2±5	8	18	13±5	18	14	16±2	-12	0	-6±6
6.1	***Plantago major***	17	9	13±4	58	56	57±1	5	7	6±1	-28	-36	-32±4	21	11	16±5	-30	-22	-26±4

6.2		22	6	14±8	26	32	29±3	9	1	5±4	-89	-105	-97±8	58	72	65±7	-47	-31	-39±8
6.3		10	12	11±1	22	28	24±4	5	-1	2±3	-114	-94	-100±10	60	64	62±2	-46	-42	-44±2
6.4		10	10	10±0	21	17	19±2	-1	1	0±1	-145	-95	-120±25	70	56	63±7	-26	-46	-36±10
6.5		-23	-27	-25±2	5	3	4±1	4	-6	-1±5	3	1	2±1	20	14	17±3	-4	-14	-9±5
7.1	***Origanum vulgare***	16	4	10±6	47	43	45±2	15	9	12±3	-25	-35	-30±5	15	11	13±2	7	13	10±3
7.2		25	17	21±4	45	39	42±3	41	41	41±0	-109	-161	-135±26	83	91	87±4	30	32	31±1
7.3		20	24	22±2	33	31	32±1	8	10	9±1	-91	-125	-108±17	78	96	87±9	26	34	30±4
7.4		12	10	11±1	31	21	26±5	3	13	8±5	-64	-48	-56±8	82	84	83±1	32	22	27±5
7.5		-21	-35	-28±7	-13	-21	-17±4	2	-2	0±2	2	4	3±1	19	15	17±2	18	14	16±2
8.1	***Dysphania ambrosioides***	8	12	10±2	77	87	82±5	15	5	10±5	-38	-54	-46±8	50	50	50±0	-3	1	-1±2
8.2		32	40	36±4	85	73	79±6	44	54	49±5	-102	-98	-100±2	98	88	93±5	-1	-9	-5±4
8.3		10	14	12±2	65	55	60±5	32	46	39±7	-60	-66	-63±3	89	89	89±0	3	1	2±1
8.4		10	8	9±1	51	55	53±2	19	-3	8±11	-83	-119	-100±18	81	85	83±2	2	10	6±4
8.5		-21	-31	-26±5	-32	-24	-28±4	2	-4	-1±3	-42	-34	-38±4	-17	-7	-12±5	41	29	35±6
9.1	***Ziziphus oxyphylla***	16	14	15±1	53	49	51±2	11	19	15±4	30	46	38±4	22	20	21±1	30	24	27±3
9.2		11	19	15±4	34	32	33±1	14	8	11±3	-93	-85	-89±4	83	89	86±3	43	53	48±5
9.3		18	22	20±2	34	36	35±1	11	9	10±1	-90	-128	-109±19	84	86	85±1	24	32	28±4
9.4		7	9	8±1	29	33	31±2	19	11	15±4	-50	-74	-62±12	78	78	78±0	11	-1	5±6
9.5		-30	-18	-24±6	5	7	6±1	-1	3	1±2	-12	-4	-8±4	24	20	22±2	-5	-5	-5±0
10.1	***Thymus linearis***	13	15	14±1	65	65	65±0	14	8	11±3	-25	-31	-28±3	23	21	22±1	8	20	14±6
10.2		25	29	27±2	63	59	61±2	34	22	28±6	-127	-137	-132±5	35	49	42±7	42	44	43±1
10.3		26	38	32±6	46	42	44±2	24	28	26±2	-74	-90	-82±8)	32	50	41±9	7	13	10±3
10.4		21	17	19±2	25	35	30±5	33	29	31±2	-108	-68	-88±20	47	51	49±2	5	-1	2±3
10.5		-16	-26	-21±5	1	3	2±1	0	2	1±1	19	19	19±0	21	29	25±4	3	-1	1±2
11.1	***Mentha longifolia***	12	16	14±2	41	49	45±4	12	6	9±3	10	16	13±3	26	10	18±8	15	17	16±1
11.2		17	11	14±3	47	43	45±2	13	11	12±1	-55	-59	-57±2	53	67	60±7	25	25	25±0
11.3		16	12	14±2	59	51	55±4	9	23	16±7	-76	-64	-70±6	81	85	83±2	-2	2	0±2
11.4		12	14	13±1	22	26	24±2	13	9	11±2	-98	-44	-71±27	17	15	16±1	14	6	10±4
11.5		-25	-27	-26±1	6	8	7±1	4	0	2±2	15	21	18±3	18	24	21±3	-15	-17	-16±1
12.1	***Punica granatum***	14	6	10±4	23	31	27±4	9	13	11±2	17	27	22±5	30	28	29±1	7	21	14±7
12.2		21	27	24±3	62	64	63±1	110	98	104±6	33	25	29±4	85	85	85±0	26	28	27±1

12.3		25	21	23±2	54	44	49±5	101	99	100±1	30	26	28±2	97	93	95±2	33	44	37±4
12.4		36	40	38±2	55	53	54±1	93	93	93±0	39	55	47±8	99	99	99±0	29	33	31±2
12.5		-22	-30	-26±4	11	17	14±3	80	70	75±5	1	11	6±5	29	27	28±1	-15	-21	-18±3
13.1	***Juglans regia***	8	10	9±1	35	49	42±7	8	12	10±2	4	6	5±1	4	0	2±2	-2	-6	-4±2
13.2		95	111	103±8	102	126	114±12	101	95	98±3	97	79	88±9	112	102	107±5	96	108	102±6
13.3		105	95	100±5	117	105	111±6	110	94	102±8	80	74	77±3	124	108	116±8	99	107	103±4
13.4		112	98	105±7	98	94	96±2	50	74	62±12	86	90	88±2	105	111	108±3	119	101	110±9
13.5		-37	-43	-40±3	7	9	8±1	7	-7	0±7	-23	-9	-16±7	14	18	16±2	41	37	39±2
14.1	***Salvia moorcroftiana***	12	14	13±1	14	18	16±2	10	6	8±2	-12	-6	-9±3	10	8	9±1	10	12	11±1
14.2		22	16	19±3	-2	-6	-4±2	8	8	8±0	-19	-5	-12±7	77	77	77±0	50	50	50±0
14.3		10	8	9±1	-19	-21	-20±1	5	3	4±1	-6	-36	-21±15	80	62	77±9	31	37	34±3
14.4		6	8	7±1	-40	-50	-45±5	2	8	5±3	-25	-43	-34±9	54	44	49±5	60	72	66±6
14.5		15	23	19±4	-8	0	-4±4	38	22	30±8	101	127	114±13	21	17	19±2	43	49	46±3
15.1	***Artemesia maritima***	21	15	18±3	63	67	65±2	24	12	18±6	8	6	7±1	8	8	8±0	-4	0	-2±2
15.2		49	45	47±2	58	54	56±2	22	26	24±2	-13	-19	-16±3	77	93	85±8	48	38	43±5
15.3		50	48	49±1	33	31	32±1	23	17	20±3	-11	-7	-9±2	78	90	84±6	60	58	59±1
15.4		6	14	10±4	31	37	34±3	18	16	17±1	-32	-38	-35±3	73	67	70±3	12	22	17±5
15.5		-19	-29	-24±5	-16	-20	-18±2	5	-3	1±4	20	10	15±5	19	17	18±1	3	5	4±1
16.1	***Mentha spicata***	15	7	11±4	1	-11	-5±6	3	7	5±2	-3	1	-1±2	-3	-11	-7±4	2	2	2±0
16.2		13	17	15±2	-3	-1	-2±1	8	8	8±0	-54	-68	-61±7	79	69	74±5	15	9	12±3
16.3		13	19	16±3	26	12	19±7	22	18	20±2	3	1	2±1	73	81	77±4	43	35	39±4
16.4		8	12	10±2	2	4	3±1	20	6	13±7	-4	-2	-3±1	60	68	64±4	52	64	58±6
16.5		-19	-33	-26±7	-15	-27	-21±6	-6	6	0±6	-2	-2	-2±0	18	14	16±2	36	38	37±1
17.1	***Nasturtium officinale***	14	6	10±4	21	13	17±4	11	7	9±2	-24	-32	-28±4	2	0	1±1	-3	-7	-5±2
17.2		44	48	46±2	27	23	25±2	9	13	11±2	-12	-30	-21±9	75	85	80±5	71	61	66±5
17.3		38	46	42±4	19	21	20±1	1	1	1±0	-34	-10	-22±12	86	82	84±2	62	64	63±1
17.4		17	21	19±2	-3	-1	-2±1	3	-3	0±3	-30	-34	-32±2	79	77	78±1	30	26	28±2
17.5		-27	-37	-32±5	-40	-32	-36±4	-1	-3	-2±1	6	-8	-1±7	16	28	22±6	-9	-23	-16±7

Legend. No. 1-17: Plants mentioned: sub-number (-1 to -5): hexane, acetone, ethanol, methanol, water; E1: Experiment 1; E2: Experiment 2; M±SD: Mean ± Standard Deviation.

S6 Table. Antibiofilm activity (percent growth inhibition of two replicate experiments, mean ± SD) of plant extracts against *Staphylococcus aureus* (USA 300).

Herb No.	Plant name	Hexane extracts			Acetone extracts			Ethanol extracts			Methanol extracts			Water extracts		
		E1	E2	M±SD	E1	E2	M±SD	E1	E2	M±SD	E1	E2	M±SD	E1	E2	M±SD
1	***Debreagesia salicifolia***	1	3	2±1	19	7	13±6	7	11	9±2	54	60	57±3	83	71	77±6
2	***Ajuga bracteosa***	15	15	15±0	81	79	80±1	97	97	97±0	23	11	17±6	50	52	51±1
3	***Berberis lycium***	31	23	27±4	23	31	115±14	123	105	114±9	137	105	121±16	35	21	28±7
4	***Aesculus indica***	66	68	67±1	66	68	86±2	77	87	82±5	18	28	23±5	24	24	24±0
5	***Calotropis procera***	15	11	13±2	92	78	85±7	139	99	119±20	94	86	90±4	96	92	94±2
6	***Plantago major***	64	74	69±5	64	74	95±5	85	91	88±3	13	15	14±1	86	76	81±5
7	***Origanum vulgare***	25	25	25±0	16	12	14±2	111	95	103±8	1	13	7±6	4	18	11±7
8	***Dysphania ambrosioides***	5	11	8±3	5	11	48±2	53	57	55±2	9	7	8±1	55	61	58±3
9	***Ziziphus oxyphylla***	16	-2	7±9	44	32	38±6	32	32	32±0	24	30	27±3	85	85	85±0
10	***Thymus linearis***	29	29	29±0	29	29	80±0	56	52	54±2	13	25	19±6	90	86	88±2
11	***Mentha longifolia***	5	21	13±8	5	21	107±9	38	50	44±6	27	19	23±4	9	19	14±5
12	***Punica granatum***	43	37	40±3	21	11	16±5	13	7	10±3	-2	12	5±7	53	39	46±7
13	***Juglans regia***	7	21	14±7	99	97	98±1	94	98	96±2	108	98	103±5	94	120	107±13
14	***Salvia moorcroftiana***	74	58	66±8	58	74	74±0	66	66	66±0	20	16	18±2	29	25	27±2
15	***Artemesia maritima***	84	76	80±4	76	84	23±7	63	73	68±5	75	75	75±0	77	85	81±4
16	***Mentha spicata***	70	90	80±10	70	90	84±5	111	93	102±9	90	82	86±4	70	70	70±0
17	***Nasturtium officinale***	69	65	67±2	82	78	80±2	107	99	103±4	98	114	106±8	12	10	11±1

Legend. E1: Experiment 1; E2: Experiment 2; M±SD: Mean ± Standard Deviation.

S7 Table. Antibiofilm activity (percent growth inhibition of two replicate experiments, mean ± SD) of plant extracts against *Candida albicans* (SC 5314).

Herb No.	*Plant name*	*Hexane extracts*			*Acetone extracts*			*Ethanol extracts*			*Methanol extracts*			*Water extracts*		
		E1	E2	M±SD	E1	E2	M±SD	E1	E2	M±SD	E1	E2	M±SD	E1	E2	M±SD
1	*Debreagesia salicifolia*	11	13	12±1	32	46	39±7	54	36	45±9	20	20	20±0	-3	13	5±8
2	*Ajuga bracteosa*	6	16	11±5	13	9	11±2	11	15	13±2	13	9	11±2	6	8	7±1
3	*Berberis lycium*	11	11	11±0	32	16	24±8	62	52	57±5	56	44	50±6	16	6	11±5
4	*Aesculus indica*	15	9	12±3	25	3	14±11	59	51	55±4	77	85	81±4	84	96	90±6
5	*Calotropis procera*	22	36	29±7	22	28	25±3	7	23	15±8	26	26	26±0	6	8	7±1
6	*Plantago major*	14	8	11±3	26	14	20±6	17	23	20±3	30	12	21±9	21	15	18±3
7	*Origanum vulgare*	16	8	12±4	13	15	14±1	17	13	15±2	7	13	10±3	19	31	25±6
8	*Dysphania ambrosioides*	9	9	9±0	8	12	10±2	6	16	11±5	8	10	9±1	27	31	29±2
9	*Ziziphus oxyphylla*	22	6	14±8	30	14	22±8	17	17	17±0	19	11	15±4	37	19	28±9
10	*Thymus linearis*	-3	23	10±13	8	36	22±14	22	18	20±2	18	30	24±6	32	22	27±5
11	*Mentha longifolia*	15	11	13±2	23	29	26±3	18	30	24±6	20	18	19±1	32	36	34±2
12	*Punica granatum*	9	21	15±6	32	24	28±4	35	13	24±11	11	19	15±4	7	7	7±0
13	*Juglans regia*	11	7	9±2	50	50	50±0	102	84	93±9	73	91	82±9	-8	22	7±15
14	*Salvia moorcroftiana*	16	6	11±5	28	14	21±7	7	21	14±7	18	12	15±3	27	15	21±6
15	*Artemesia maritima*	18	34	26±8	63	63	63±0	89	81	85±4	65	73	69±4	26	18	22±4
16	*Mentha spicata*	17	5	11±6	22	16	19±3	31	31	31±0	34	34	34±0	51	51	51±0
17	*Nasturtium officinale*	7	13	10±3	7	25	16±9	19	31	25±6	42	28	35±7	51	65	58±7

Legend. E1: Experiment 1; E2: Experiment 2; M±SD: Mean ± Standard Deviation.

S8 Table. Cytotoxicity (cell viability inhibition of two replicate experiments, mean ± SD) of plant extracts against A549 cell lines.

Herb No.	*Plant name*	*Hexane extracts*			*Acetone extracts*			*Ethanol extracts*			*Methanol extracts*			*Water extracts*		
		E1	E2	M±SD	E1	E2	M±SD	E1	E2	M±SD	E1	E2	M±SD	E1	E2	M±SD
1	***Debreagesia salicifolia***	36	48	42±6	47	55	51±4	45	31	38±7	52	34	43±9	11	13	12±1
2	***Ajuga bracteosa***	45	31	38±7	59	47	53±6	41	49	45±4	40	50	45±5	-12	-10	-11±1
3	***Berberis lycium***	33	51	42±9	23	9	16±7	24	36	30±6	20	38	29±9	16	14	15±1
4	***Aesculus indica***	-3	7	2±5	53	63	58±5	91	89	90±1	90	90	90±0	-9	-5	-7±2
5	***Calotropis procera***	61	47	54±7	16	30	23±7	14	26	20±6	32	14	23±9	6	2	4±2
6	***Plantago major***	-3	-7	-5±2	20	8	14±6	8	14	11±3	57	43	50±7	-14	-12	-13±1
7	***Origanum vulgare***	36	40	38±2	-5	3	-1±4	-1	-3	-2±1	12	6	4±2	-4	-6	-5±1
8	***Dysphania ambrosioides***	-5	1	-2±3	8	2	5±3	27	17	22±5	38	22	30±8	-12	-8	-10±2
9	***Ziziphus oxyphylla***	46	46	46±0	28	12	20±8	27	17	22±5	19	31	25±6	18	14	16±2
10	***Thymus linearis***	1	-3	-1±2	3	5	4±1	9	15	13±3	33	55	42±9	-14	-14	-14±0
11	***Mentha longifolia***	41	41	41±0	12	2	7±5	-2	4	1±3	18	4	11±7	-19	-19	-19±0
12	***Punica granatum***	-1	-1	-1±0	-8	2	-3±5	7	3	5±2	45	43	44±1	6	4	5±1
13	***Juglans regia***	36	46	41±5	-1	5	2±3	1	3	2±1	3	7	5±2	-9	-7	-8±1
14	***Salvia moorcroftiana***	-2	4	1±3	44	26	35±9	63	53	58±5	74	64	69±5	11	11	11±0
15	***Artemesia maritima***	45	43	44±1	15	27	21±6	20	12	16±4	17	15	16±1	-12	-8	-10±2
16	***Mentha spicata***	-1	-7	-4±3	10	6	8±2	15	25	20±5	52	56	54±2	12	6	9±3
17	***Nasturtium officinale***	28	28	28±0	15	25	20±5	20	18	19±1	2	8	5±3	-14	-2	-8±6

Legend. E1: Experiment 1; E2: Experiment 2; M±SD: Mean ± Standard Deviation.

S9 Table. Cytotoxicity (cell viability inhibition of two replicate experiments, mean ± SD) of plant extracts against WI-26 VA4 cell lines.

Herb No.	Plant name	Hexane extracts			Acetone extracts			Ethanol extracts			Methanol extracts			Water extracts		
		E1	E2	M±SD	E1	E2	M±SD	E1	E2	M±SD	E1	E2	M±SD	E1	E2	M±SD
1	***Debreagesia salicifolia***	51	57	54±3	61	53	57±4	63	59	61±2	52	34	37±4	11	13	12±1
2	***Ajuga bracteosa***	50	52	51±1	50	50	50±0	47	41	44±3	40	50	42±7	-12	-10	-11±1
3	***Berberis lyceum***	35	23	29±6	15	19	17±2	9	25	17±8	20	38	25±3	16	14	15±1
4	***Aesculus indica***	5	3	4±1	19	27	23±4	68	80	74±6	90	90	79±2	-9	-5	-7±2
5	***Calotropis procera***	49	53	51±2	39	27	33±6	22	12	17±5	32	14	24±5	6	2	4±2
6	***Plantago major***	5	1	3±2	-5	-17	-11±6	-6	-4	-5±1	57	43	43±4	-14	-12	-13±1
7	***Origanum vulgare***	29	35	32±3	3	5	4±1	-6	-12	-9±3	12	6	-11±3	-4	-6	-5±1
8	***Dysphania ambrosioides***	6	4	5±1	2	2	2±0	8	20	14±6	38	22	42±3	-12	-8	-10±2
9	***Ziziphus oxyphylla***	39	47	43±4	19	29	24±5	19	13	16±3	19	31	16±3	18	14	16±2
10	***Thymus linearis***	-6	-8	-7±1	-4	-8	-6±2	-3	7	2±5	33	55	38±4	-14	-14	-14±0
11	***Mentha longifolia***	32	36	34±2	11	17	14±3	-9	-13	-11±2	18	4	-12±5	-19	-19	-19±0
12	***Punica granatum***	-2	4	1±3	6	4	5±1	-2	-8	-5±3	45	43	24±4	6	4	5±1
13	***Juglans regia***	39	29	34±5	10	16	13±3	4	8	6±2	3	7	11±4	-9	-7	-8±1
14	***Salvia moorcroftiana***	2	4	3±1	9	17	13±4	27	19	23±4	74	64	35±2	11	11	11±0
15	***Artemesia maritima***	69	57	63±6	50	34	42±8	38	40	39±1	17	15	34±6	-12	-8	-10±2
16	***Mentha spicata***	-7	-5	-6±1	1	5	3±2	10	6	8±2	52	56	29±3	12	6	9±3
17	***Nasturtium officinale***	38	40	39±1	11	7	9±2	18	4	11±7	2	8	-3±1	-14	-2	-8±6

Legend. E1: Experiment 1; E2: Experiment 2; M±SD: Mean ± Standard Deviation.
