# Supplementary material for: Ethnomedicinal documentation, phytochemical characterization, and biological evaluation of the traditional medicinal plants from Swat region of Pakistan
Source: PLoS One. 2025 Aug 21;20(8):e0329735. doi: 10.1371/journal.pone.0329735 (PMC12370205; doi:10.1371/journal.pone.0329735)

Ethnobotanical data

S1 Data: Raw data for Fig 2A

Urticaceae	1
Labiatae	3
Berberidaceae	1
Hippocastanaceae	1
Asclepiadacea	1
Plantaginaceae	1
Rhamnaceae	1
Lamiaceae	3
Punicaceae	1
Juglandaceae	1
Asteraceae	1
Brassicaceae	1

S2 Data: Raw data for Fig 2B

Respiratory infection	15
Gastrointestinal infection	13
Wound infection	9
Skin infection	8
Fever	4
Liver complaints	2
Ulcerative colitis	2
Urinary infection	2
Throat infection	2
Vein complications	2
Othe infections	1

S3 Data: Raw data for Fig 2C

Leaves	10
Aerial parts	3
Root bark	2
Fruit	1
Whole plant	1

Antimicrobial data

S4 Data: Raw data for Fig 3

	SA	*AH*	*ML*	*PA*	*AB*	*EC*	*BD*	*SLE*	*EA*	*SE*	*SF*	*SS*	*STF*	*EF*	*CA*	*CAU*	*CG*	*CU*	*CP*	*SC*
1.1	25	97	45	6	9	23	97	42	16	41	42	35	1	45	14	30	12	98	10	-22
1.2	36	99	76	-44	-13	15	108	28	21	34	23	-1	3	47	19	40	71	-41	83	-15
1.3	38	99	63	-45	-14	26	91	17	4	40	1	64	33	68	21	30	15	3	38	1
1.4	70	98	86	6	35	43	100	37	74	49	12	2	63	65	29	39	10	-47	47	24
1.5	-27	-95	-28	-118	-8	-47	-157	-30	-74	-49	63	-159	48	2	-29	-5	-6	-8	17	-6
2.1	36	68	39	30	-20	1	98	23	26	6	-4	-50	-7	-13	12	28	11	-41	8	-33
2.2	23	90	46	1	5	23	100	25	19	16	-7	18	-5	-30	11	37	13	-60	48	-21
2.3	10	97	54	-7	-6	21	89	16	24	6	-17	21	-6	-64	7	35	9	-45	66	-38
2.4	-20	4	-4	-15	-49	6	60	9	9	-15	12	-5	-19	-24	11	2	-3	-56	36	-15
2.5	-16	-69	-65	-127	-41	-80	-122	14	-49	20	18	-45	-85	-35	-26	11	0	18	27	-10
3.1	1	2	55	13	11	2	22	9	25	-15	14	11	-3	43	4	3	3	-15	4	-19
3.2	97	7	99	64	18	42	100	23	26	36	27	40	19	7	15	68	93	-2	91	93
3.3	96	13	99	43	42	39	21	19	27	31	28	91	8	-6	36	63	98	4	97	97
3.4	100	40	99	30	13	34	94	19	15	29	30	47	3	-10	54	83	98	24	99	96
3.5	8	-83	34	-69	43	-53	-24	71	53	63	26	16	-59	-9	-27	29	28	45	21	-8
4.1	46	100	60	26	21	36	116	30	7	40	33	39	13	18	17	36	14	6	26	-13
4.2	68	100	81	-18	-16	33	70	27	13	36	-1	61	2	31	34	18	12	-100	36	8
4.3	18	100	84	-35	-29	29	77	13	21	12	3	54	-1	7	26	54	8	-34	41	77
4.4	63	99	101	-39	-6	13	101	16	16	19	16	42	22	39	47	55	96	6	36	94
4.5	59	-12	83	-52	-24	-53	29	29	-27	81	-4	-38	77	-15	-13	65	7	-14	26	1
5.1	-12	102	29	-9	-12	-8	84	-12	9	-3	2	24	-17	-21	12	42	19	-78	60	-36
5.2	95	102	39	4	3	28	93	1	10	14	12	-30	1	-90	14	43	10	-85	83	-25
5.3	96	100	50	-9	-26	4	77	4	-4	10	14	-44	-8	67	9	30	4	-66	66	-45
5.4	16	98	29	-64	-31	10	109	60	10	10	22	27	-7	35	11	29	5	-21	83	-7
5.5	-28	-28	-31	-82	81	-81	-179	69	-86	90	77	-84	-4	-60	-24	-17	2	13	16	-6
6.1	12	100	36	33	4	7	85	26	26	11	19	23	-9	-53	13	57	6	-32	16	-26
6.2	51	80	67	-31	0	18	88	17	6	29	14	-47	-13	-63	14	29	5	-97	65	-39
6.3	17	96	49	-32	-27	7	94	4	1	11	7	-47	-42	-121	11	24	2	-104	62	-44

6.4	-27	105	41	-52	-24	9	79	-1	-3	20	9	-53	-40	-121	10	19	0	-120	63	-36
6.5	-24	-107	-27	-125	80	-83	-133	-28	64	1	-37	-112	12	-58	-25	4	-1	2	17	-9
7.1	100	96	52	9	-3	28	44	89	6	18	5	51	0	-13	10	45	12	-30	13	10
7.2	103	95	32	-30	27	-6	43	23	-8	30	9	4	10	31	21	42	41	-135	87	31
7.3	61	113	101	-39	-10	-3	32	16	14	17	15	-1	-1	-47	22	32	9	-108	87	30
7.4	-95	101	78	-51	-11	15	75	15	8	-3	12	-6	5	-23	11	26	8	-56	83	27
7.5	-8	-91	-40	-128	-44	-87	-118	67	-87	-13	-61	-101	-135	35	-28	-17	0	3	17	16
8.1	28	56	53	6	-19	2	83	19	9	20	8	23	1	2	10	82	10	-46	50	-1
8.2	99	97	83	-22	19	18	93	30	-13	34	12	54	-8	104	36	79	49	-100	93	-5
8.3	100	99	74	-33	-1	6	93	12	11	30	9	46	13	10	12	60	39	-63	89	2
8.4	-27	36	35	-25	-12	18	83	17	6	9	-1	16	-14	-55	9	53	8	-101	83	6
8.5	-45	-102	-75	-119	99	-67	-148	90	-90	7	81	-89	-133	39	-26	-28	-1	-38	-12	35
9.1	65	109	60	22	-1	27	27	30	8	31	18	17	10	-68	15	51	15	38	21	27
9.2	102	100	80	18	23	14	104	24	27	42	-2	7	11	76	15	33	11	-89	86	48
9.3	99	98	59	9	22	19	89	24	21	34	-8	4	24	100	20	35	10	-109	85	28
9.4	-23	97	35	-12	-7	-10	82	12	3	7	11	-3	7	95	8	31	15	-62	78	5
9.5	-20	-77	8	-137	-47	-52	-125	-17	-57	-85	-44	6	-77	46	-24	6	1	-8	22	-5
10.1	27	56	54	48	24	-17	79	-4	6	6	20	27	8	-26	14	65	11	-28	22	14
10.2	80	96	93	-51	5	16	104	38	9	44	9	36	12	-78	27	61	28	-132	42	43
10.3	98	101	87	-10	8	12	128	25	10	41	16	51	5	75	32	44	26	-82	41	10
10.4	97	105	89	-38	-21	17	101	17	4	38	10	23	11	66	19	30	31	-88	49	2
10.5	44	-107	-32	-138	81	-98	-69	54	-74	4	127	-55	89	0	-21	2	1	19	25	1
11.1	101	95	49	29	-1	4	81	2	4	12	14	33	4	-4	14	45	9	13	18	16
11.2	97	103	97	-48	37	26	108	26	-15	36	29	15	27	88	14	45	12	-57	60	25
11.3	99	100	85	-49	57	7	65	11	-21	27	27	-11	16	80	14	55	16	-70	83	0
11.4	-44	87	44	-24	-37	-5	86	19	19	-13	22	3	-3	-90	13	24	11	-71	16	10
11.5	22	-73	-43	-121	-40	-80	-108	-27	-77	18	-13	-85	-4	-70	-26	7	2	18	21	-16
12.1	1	36	54	29	52	8	51	11	15	35	11	2	11	-35	10	27	11	22	29	14
12.2	71	110	89	87	66	31	106	26	13	44	49	39	40	96	24	63	104	29	85	27
12.3	70	106	88	53	73	44	95	11	15	39	39	28	58	31	23	49	100	28	95	37
12.4	88	102	88	56	56	31	76	31	34	61	44	25	60	85	38	54	93	47	99	31
12.5	71	99	60	-3	11	-28	1	5	-1	92	78	14	31	24	-26	14	75	6	28	-18
13.1	101	99	38	28	3	17	89	19	7	17	17	49	24	-74	9	42	10	5	2	-4

13.2	91	91	120	71	123	91	90	42	25	97	100	100	101	146	103	114	98	88	107	102
13.3	92	91	89	97	91	78	92	50	29	126	103	99	86	92	100	111	102	77	116	103
13.4	103	96	99	90	90	81	96	53	30	96	102	100	102	99	105	96	62	88	108	110
13.5	95	97	57	-25	12	-30	29	2	2	96	25	-5	-5	10	-40	8	0	-16	16	39
14.1	94	46	42	9	-15	-26	66	5	6	-9	-1	32	9	-173	13	16	8	-9	9	11
14.2	100	96	59	-5	6	-7	89	26	1	36	12	21	10	-42	19	-4	8	-12	77	50
14.3	-7	69	65	-6	-1	22	70	21	2	21	-13	37	5	-39	9	-20	4	-21	71	34
14.4	-61	88	46	-36	4	-9	96	19	-8	11	6	28	-4	-162	7	-45	5	-34	49	66
14.5	-13	-19	-65	19	40	-74	8	67	12	86	76	96	78	30	19	-4	30	114	19	46
15.1	98	100	60	18	6	29	90	17	7	17	20	5	13	-75	18	65	18	7	8	-2
15.2	100	105	76	-8	18	4	116	35	-8	44	22	80	20	88	47	56	24	-16	85	43
15.3	97	94	67	-1	18	4	107	26	-19	83	24	51	20	50	49	32	20	-9	84	59
15.4	97	87	69	-10	13	0	117	21	-26	35	22	86	42	76	10	34	17	-35	70	17
15.5	-23	-96	-29	-132	77	-85	-114	-25	84	-6	-33	-91	-14	-68	-24	-18	1	15	18	4
16.1	100	24	41	28	2	13	-7	31	12	32	18	4	22	-162	11	-5	5	-1	-7	2
16.2	101	56	79	-3	-19	0	73	29	-5	27	8	27	14	1	15	-2	8	-61	74	12
16.3	95	79	63	-58	-41	6	82	29	14	32	15	-20	16	-64	16	19	20	2	77	39
16.4	99	91	48	-18	-16	7	87	28	18	29	24	-20	18	-43	10	3	13	-3	64	58
16.5	-15	-88	-38	17	-27	-92	-100	-23	-82	2	-31	-90	75	-90	-26	-21	0	-2	16	37
17.1	100	57	33	44	17	29	41	10	22	32	-7	41	22	-150	10	17	9	-28	1	-5
17.2	106	94	77	-10	18	3	100	28	36	82	27	101	23	77	46	25	11	-21	80	66
17.3	93	97	49	4	46	64	91	33	11	28	18	17	20	67	42	20	1	-22	84	63
17.4	66	33	41	21	4	24	84	17	26	73	6	70	9	56	19	-2	0	-32	78	28
17.5	-30	-60	-19	-101	-54	-96	-128	-28	-99	-33	-20	-38	5	34	-32	-36	-2	-1	22	-16

Antimicrobial data

S5 Data: Raw data for Fig 4A

Debreagesia salicifolia	21
Ajuga bracteosa	9
Berberis lycium	27
Aesculus indica	26
Calotropis procera	21
Plantago major	17
Origanum vulgare	17
Dysphania ambrosioides	26
Ziziphus oxyphylla	20
Thymus linearis	25
Mentha longifolia	19
Punica granatum	41
Juglans regia	63
Salvia moorcroftiana	21
Artemesia maritima	31
Mentha spicata	17
Nasturtium officinale	25

S6 Data: Raw data for Fig 4B

SA	47
AH	60
ML	50
PA	7
AB	13
EC	4
BD	60
SE	10
EA	4
SE	13
SF	10
SS	16
STF	10
EF	22
CA	4
CAU	22
CG	13
CU	5
CP	42
SC	14

S7 Data: Raw data for Fig 4C

Hexane extracts	Number of	Percentage
Acetonitrile extracts	52	12
Ethanol extracts	115	27
Methanol extracts	116	27
Water extracts	101	24
	42	10

Antibiofilm data

S8 Data: Raw data for Fig 5A

Herb No.	H-USA 300	A-USA 300	E-USA 300	M-USA 30(	W-USA 30(	H-SC 5314	A-SC 5314	E-SC 5314	M-SC 5314	W-SC 5314
1	2	13	9	57	77	12	39	45	20	5
2	15	80	97	17	51	11	11	13	11	7
3	27	115	114	121	28	11	24	57	50	11
4	67	86	82	23	24	12	14	55	81	90
5	13	85	119	90	94	29	25	15	26	7
6	69	92	88	14	81	11	20	20	21	18
7	25	14	103	7	11	12	14	15	10	25
8	8	48	55	8	58	9	10	11	9	29
9	7	38	32	27	85	14	22	17	15	28
10	29	80	54	19	88	10	22	20	24	27
11	13	107	44	23	14	13	26	24	19	34
12	40	16	10	5	46	15	28	24	15	7
13	14	98	96	103	107	9	50	93	82	7
14	66	74	66	18	27	11	21	14	15	21
15	80	23	68	75	81	26	63	85	69	22
16	80	84	102	86	70	11	19	31	34	51
17	67	80	103	106	11	10	16	25	35	58

Antibiofilm data

S9 Data: Raw data for Fig 5B

	SA (USA 300)	CA (SC 5314)
Hexane	13	0
Acetone	23	15
Ethanol	28	31
Methanol	15	31
Water	21	23

S10 Data: Data Fig 5C

		SA (USA 300)	CA (SC 5314)
1	*Debreagesia salicifolia*	2	0
2	*Ajuga bracteosa*	3	0
3	*Berberis lycium*	3	2
4	*Aesculus indica*	3	3
5	*Calotropis procera*	4	0
6	*Plantago major*	4	0
7	*Origanum vulgare*	1	0
8	*Dysphania ambrosioides*	2	0
9	*Ziziphus oxyphylla*	1	0
10	*Thymus linearis*	3	0
11	*Mentha longifolia*	1	0
12	*Punica granatum*	0	0
13	*Juglans regia*	4	3
14	*Salvia moorcroftiana*	3	0
15	*Artemesia maritima*	4	3
16	*Mentha spicata*	5	1
17	*Nasturtium officinale*	4	1

Cytotoxicity data

S11 Data: Raw data for Fig 6A

	H-A549	A-A549	E-A549	M-A549	W-A549	H-W-26 VA4	A-WI-26 VA	E-WI-26 VA	M-WI-26 VA	V-WI-26 VA4
1	42	51	38	43	12	54	57	61	37	15
2	38	53	45	45	-11	51	50	44	42	10
3	42	16	30	29	15	29	17	17	25	-9
4	2	58	90	90	-7	4	23	74	79	17
5	54	23	20	23	4	51	33	17	24	6
6	-5	14	11	50	-13	3	-11	-5	43	-10
7	38	-1	-2	4	-5	32	4	-9	-11	-19
8	-2	5	22	30	-10	5	2	14	42	8
9	46	20	22	25	-16	43	24	16	16	17
10	-1	4	13	42	-14	-7	-6	2	38	-11
11	41	7	1	11	-19	34	14	-11	-12	14
12	-1	-3	5	44	5	1	5	-5	24	10
13	41	2	2	5	-8	34	13	6	11	-5
14	1	35	58	69	11	3	13	23	35	16
15	44	21	16	16	-10	63	42	39	34	13
16	-4	8	20	54	9	-6	3	8	29	20
17	28	20	19	5	-8	39	9	11	-3	-8

Cytotoxicity data

S12 Data: Raw data for Fig 6B

		A549	WI-26 VA4
1	Debreagesia salicifolia	10	33
2	Ajuga bracteosa	10	22
3	Berberis lycium	0	0
4	Aesculus indica	30	22
5	Calotropis procera	10	11
6	Plantago major	10	0
7	Origanum vulgare	0	0
8	Dysphania ambrosioides	0	0
9	Ziziphus oxyphylla	0	0
10	Thymus linearis	0	0
11	Mentha longifolia	0	0
12	Punica granatum	0	0
13	Juglans regia	0	0
14	Salvia moorcroftiana	20	0
15	Artemesia maritima	0	11
16	Mentha spicata	10	0
17	Nasturtium officinale	0	0

S13 Data: Raw data for Fig 6C

	A549	WI-26 VA4
H	10	44
A	30	22
E	20	22
M	40	11
W	0	0

S14 DATA: Photographs of the plant samples for phytochemical analysis (Table 2)

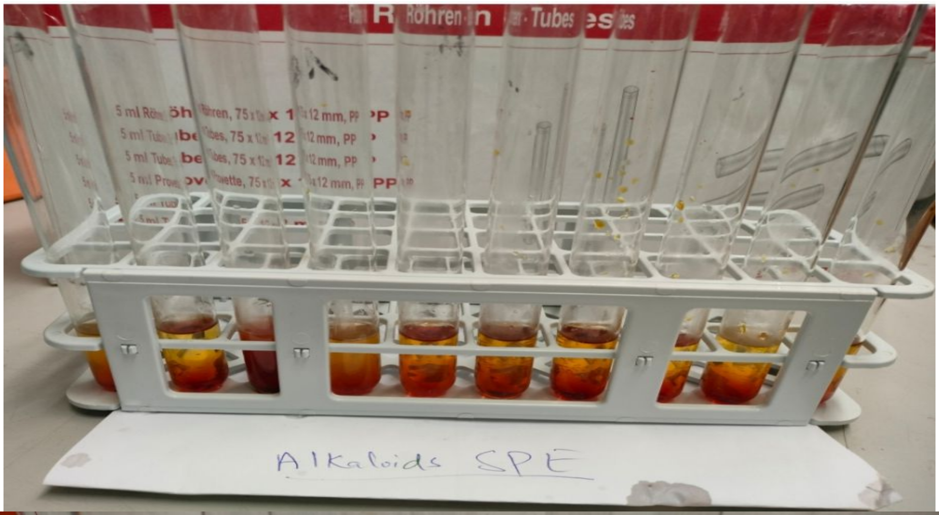

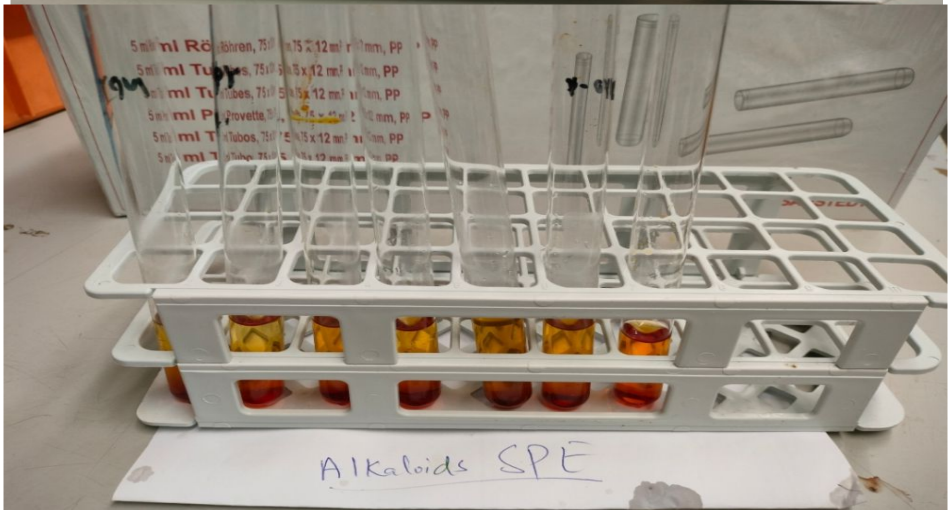

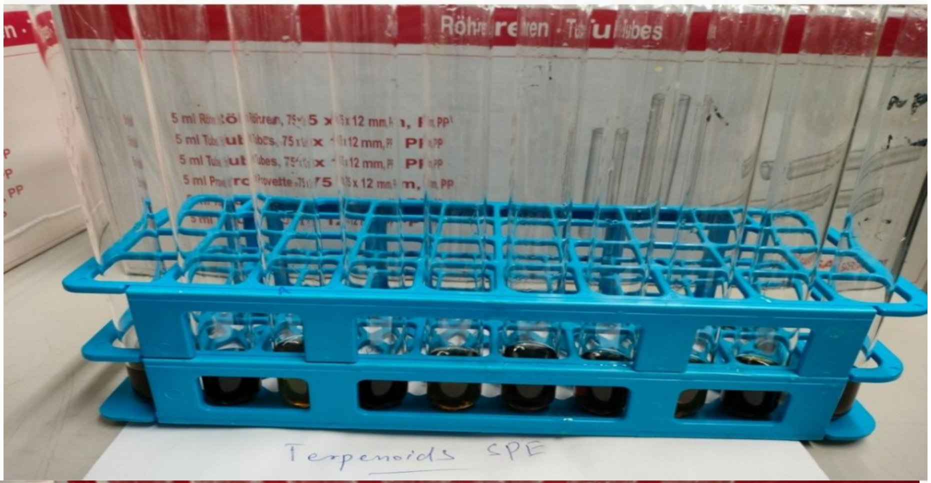

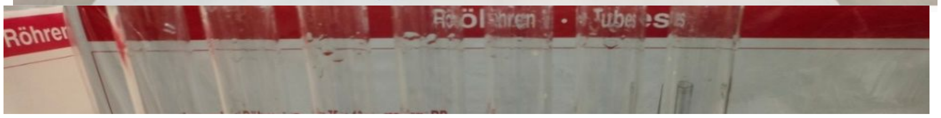

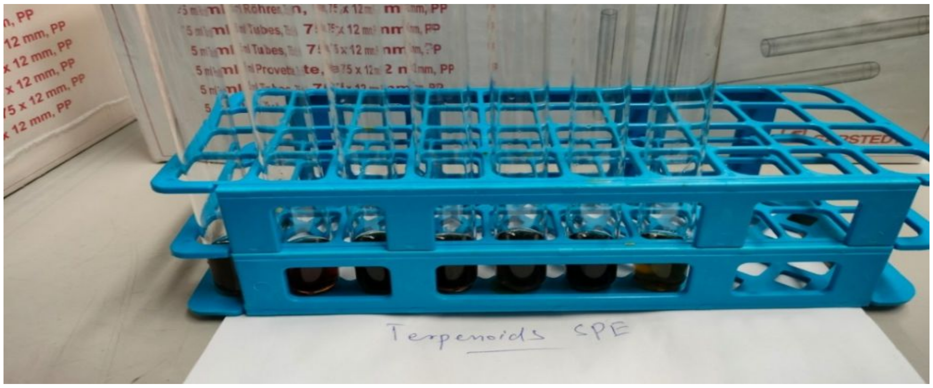
Terpenoids SPE

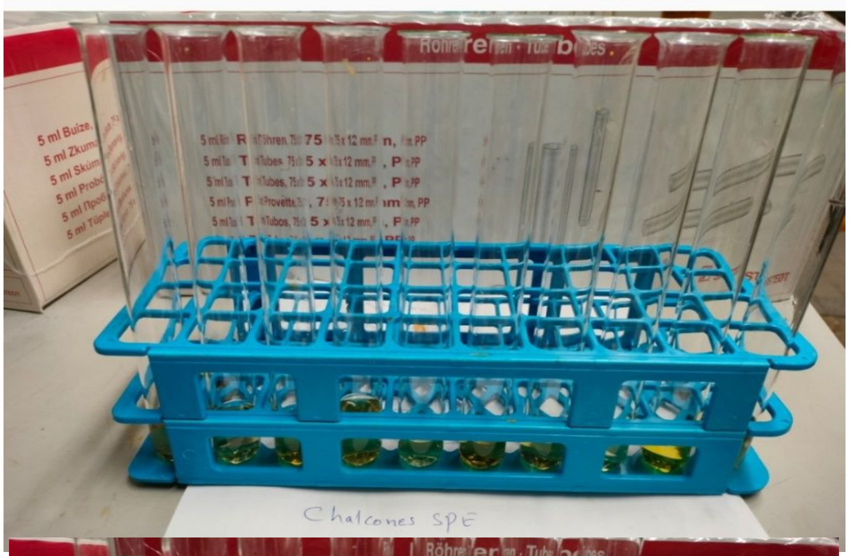
Chalcones SPE

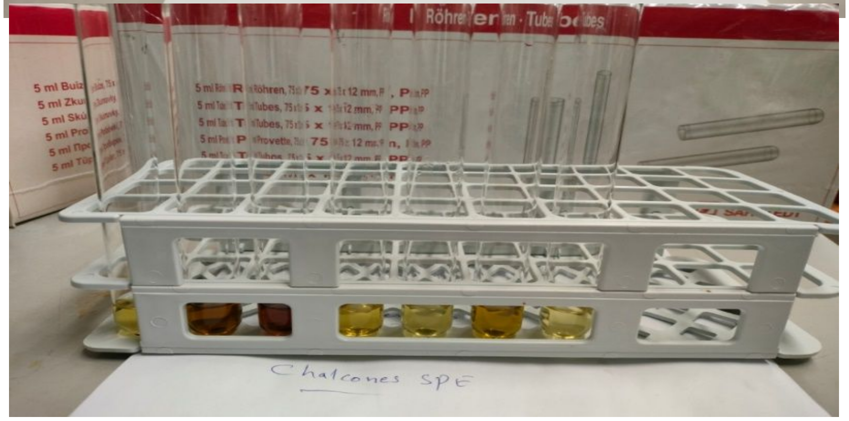
Chalcones SPE

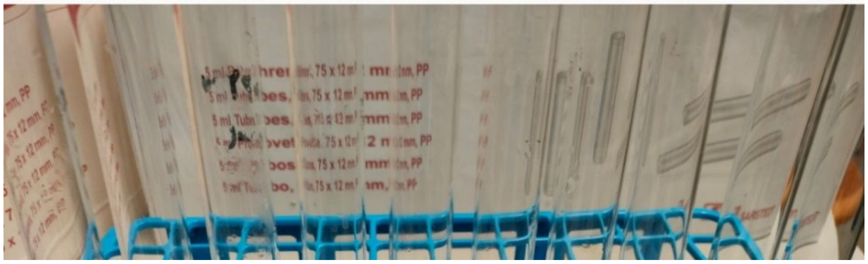

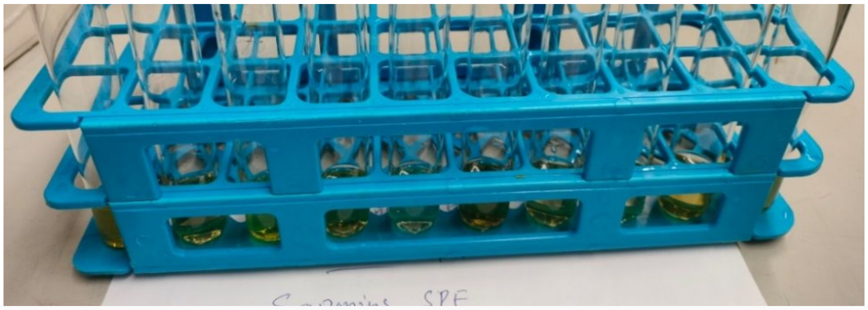

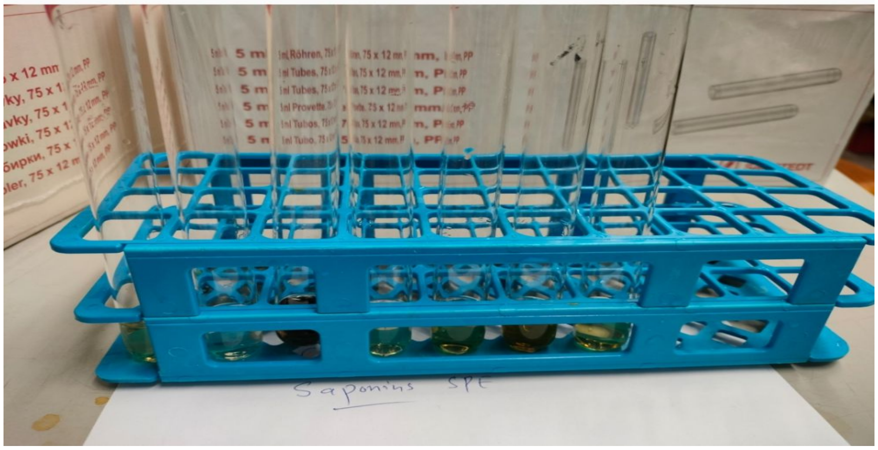
Saponins SPE

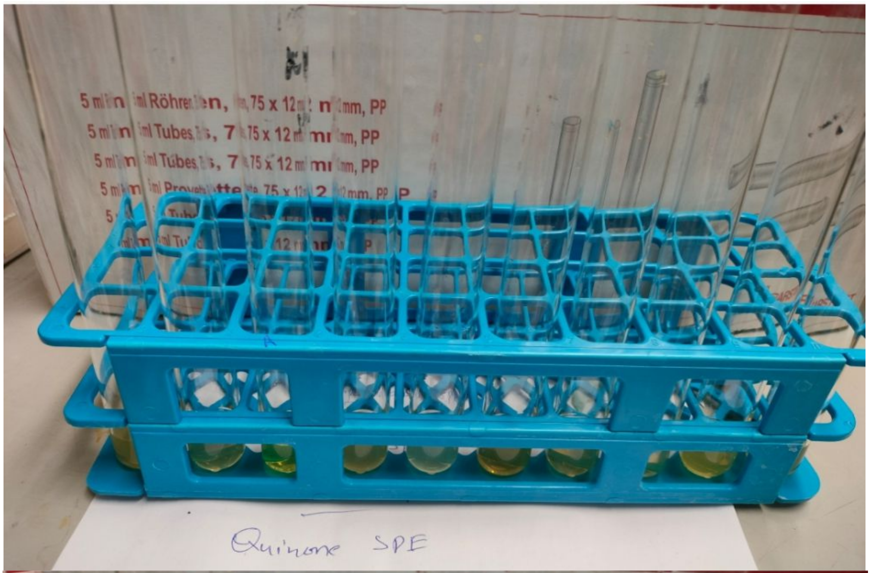
Quinone SPE

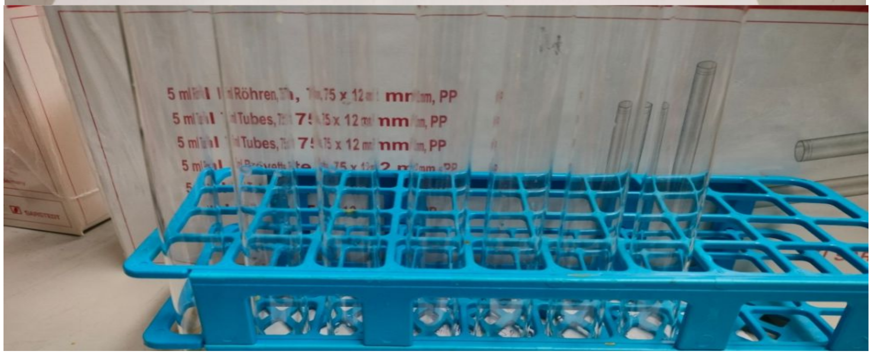

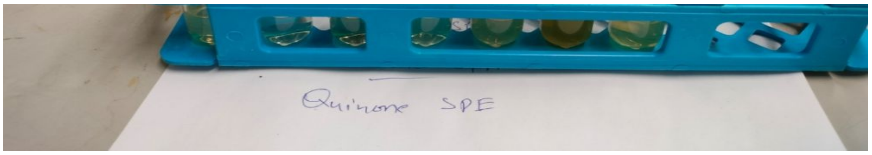
Quinone SPE

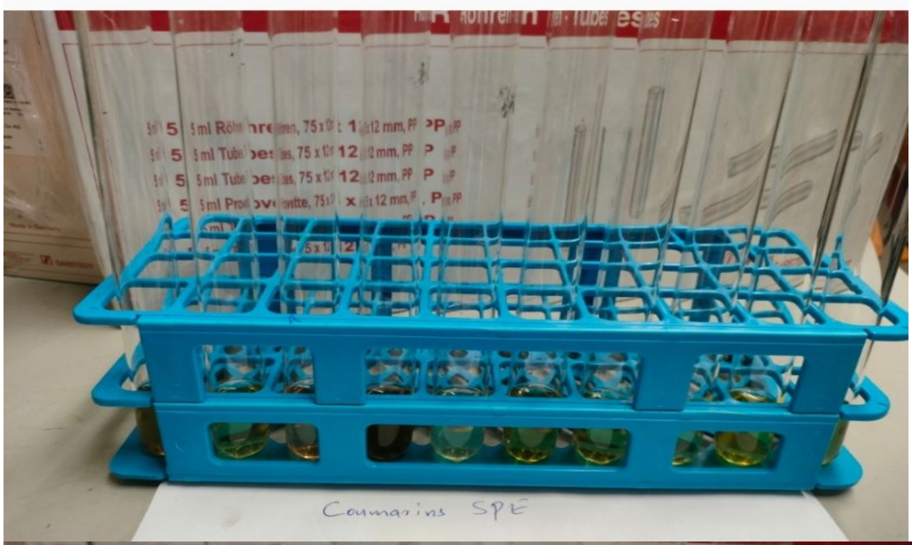
Coumarins SPE

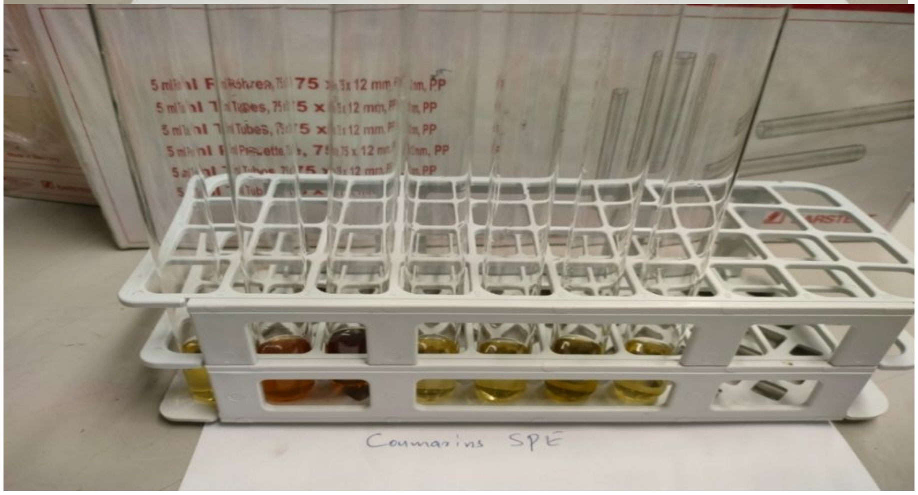
Coumarins SPE

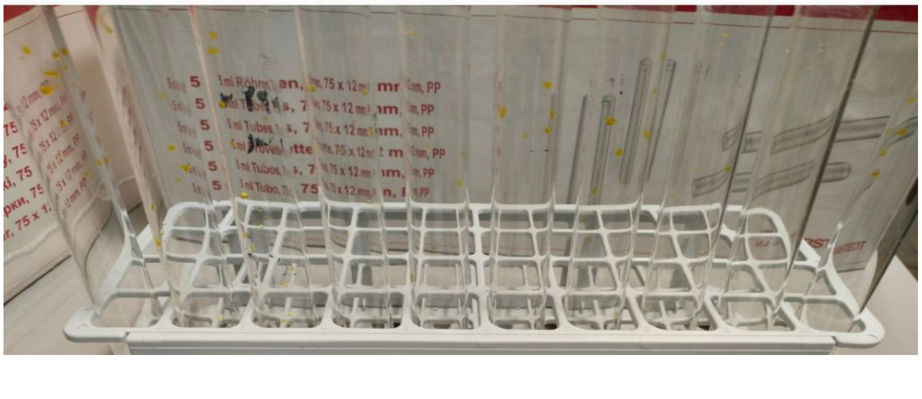

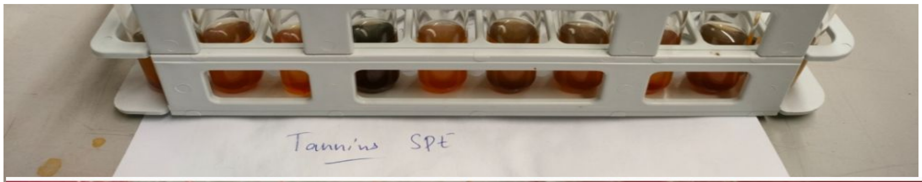
Tannins SPE

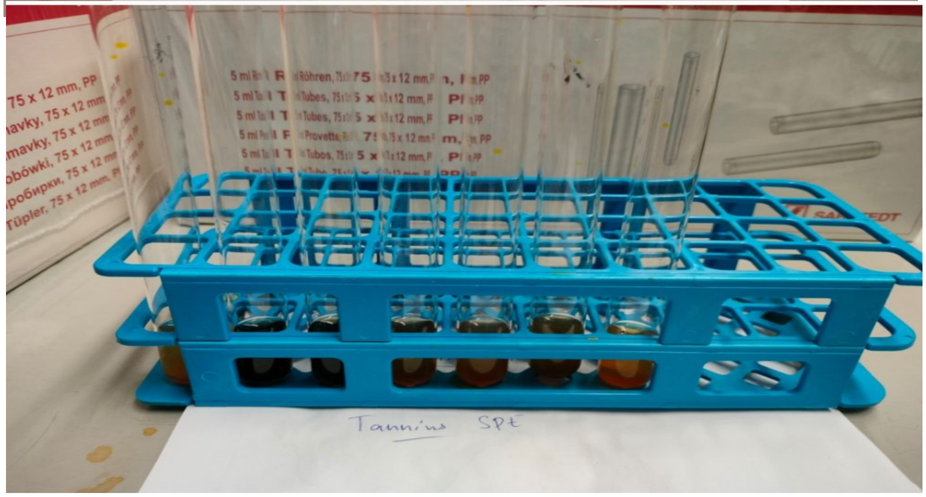
Tannins SPE

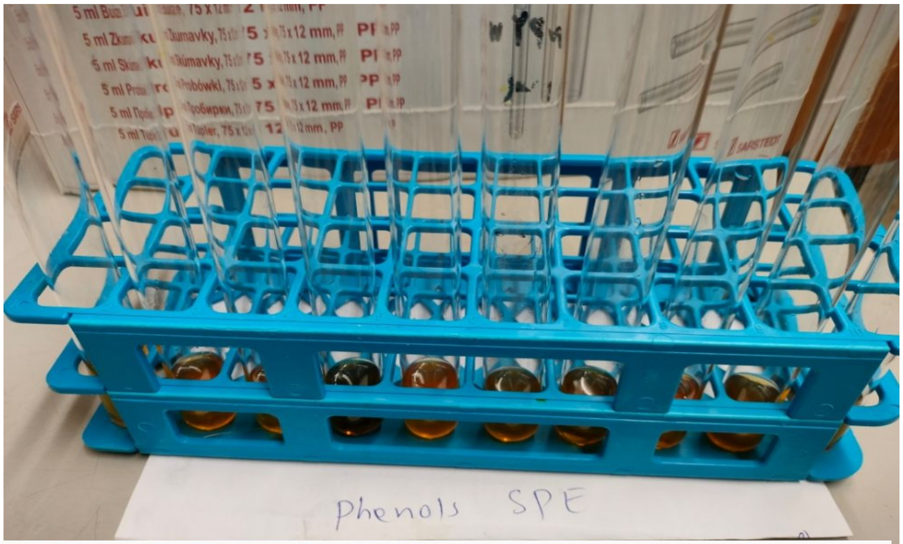
Phenols SPE

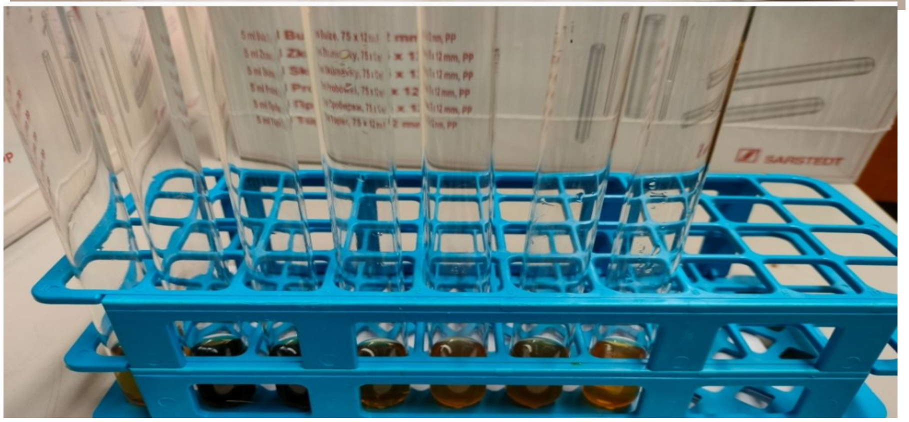

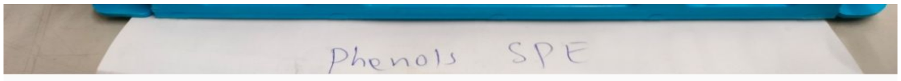
Phenols SPE

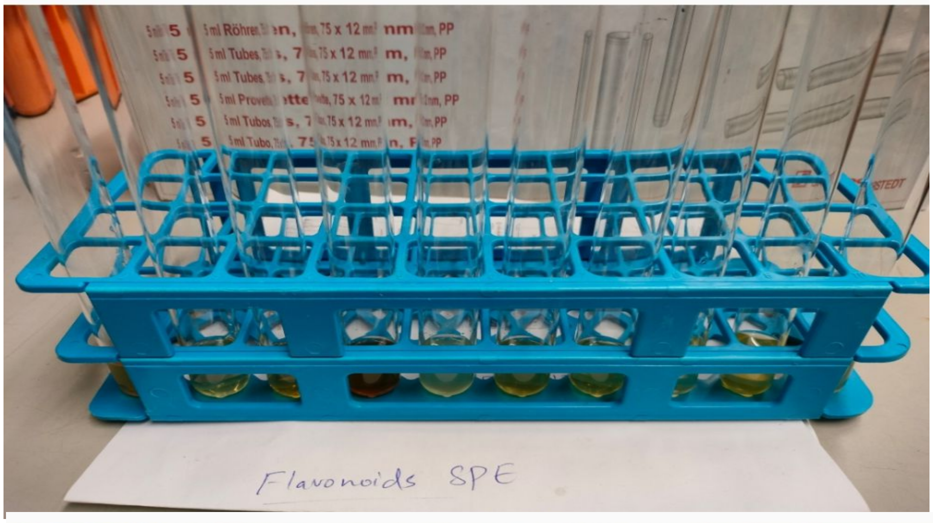
Flavonoids SPE

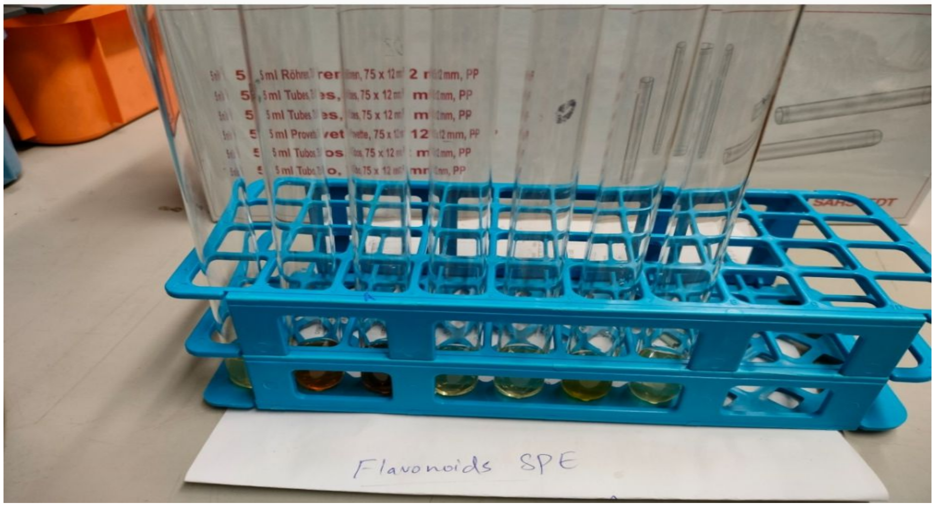
Flavonoids SPE

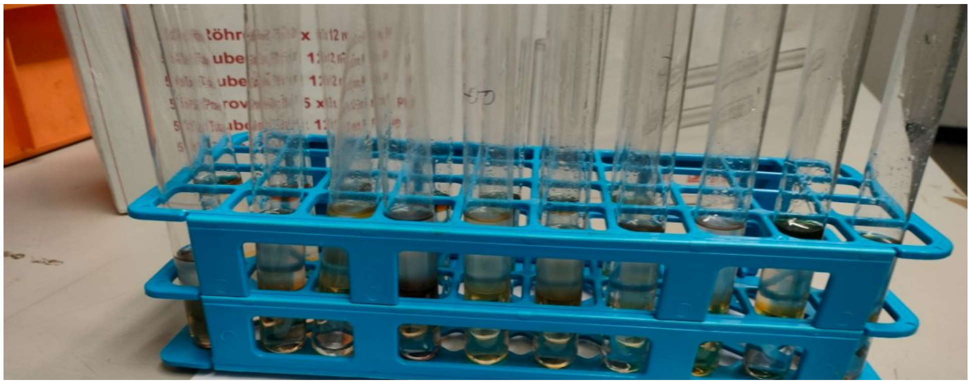

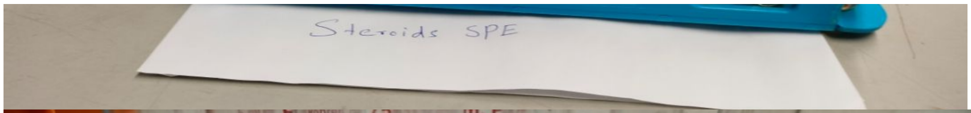
Steroids SPE

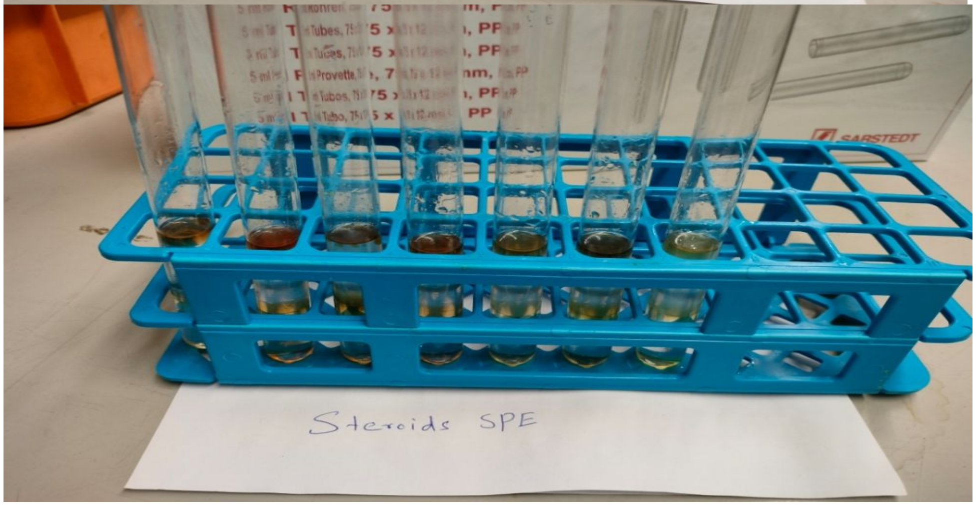
SARSTEDT
Steroids SPE

S15 Data: Photographic images of TLC fingerprints corresponding to Table 3

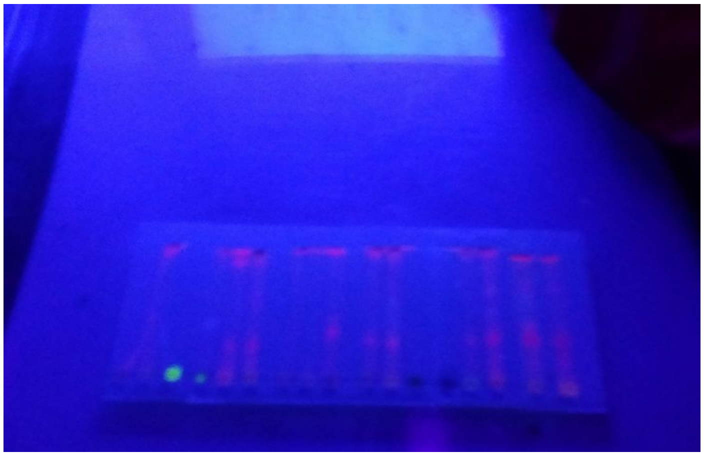

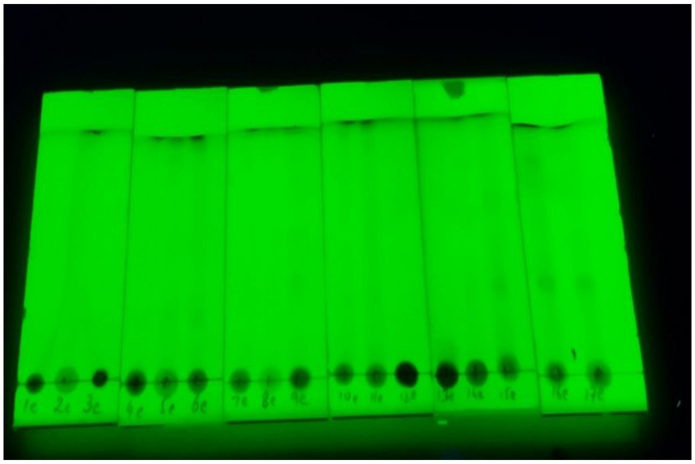

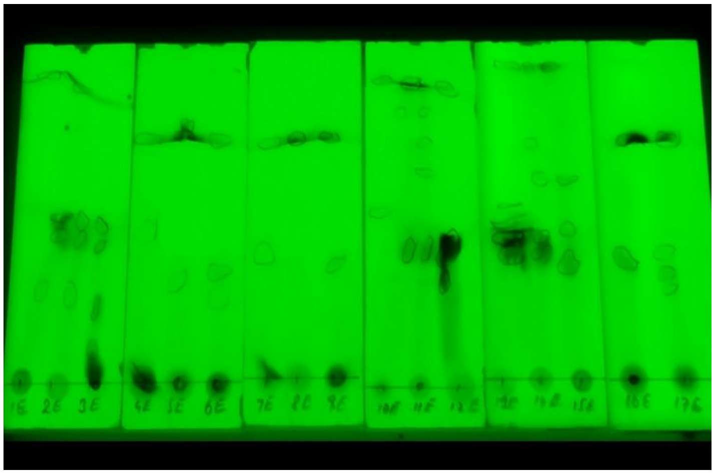

Supplement: S2 File — Raw data corresponding to Figs 2–6 and selected tables. S1 Data. Raw data for Fig 2A. S2 Data. Raw data for Fig 2B. S3 Data. Raw data for Fig 2C. S4 Data. Raw data for Fig 3. S5 Data. Raw data for Fig 4A. S6 Data. Raw data for Fig 4B. S7 Data. Raw data for Fig 4C. S8 Data. Raw data for Fig 5A. S9 Data. Raw data for Fig 5B. S10 Data. Raw data for Fig 5C. S11 Data. Raw data for Fig 6A. S12 Data. Raw data for Fig 6B. S13 Data. Raw data for Fig 6C. S14 Data. Photographs of the plant samples for phytochemical analysis (Table 2). S15 Data. Photographic images of TLC fingerprints corresponding to Table 3. (PDF) [file pone.0329735.s002.pdf]
